# ABA-GA antagonism and modular gene networks cooperatively drive acquisition of desiccation tolerance in perilla seeds

**DOI:** 10.3389/fpls.2025.1624742

**Published:** 2025-07-23

**Authors:** Xiaohuan Yang, Minghao Chen, Mingwang Liu, Bowen Li, Zhichao Sun, Ailian Lu, Sen Zhang, Xinghai Shi, Jun Ren, Xiuzhen Qin, Jinhu Ma

**Affiliations:** ^1^ College of Agriculture, Shanxi Agricultural University, Taigu, Shanxi, China; ^2^ Key Laboratory of Plant Molecular Physiology, Institute of Botany, Chinese Academy of Sciences, Beijing, China; ^3^ University of Chinese Academy of Sciences, Beijing, China; ^4^ School of Innovation and Intrepreneurship, Shanxi Agricultural University, Taigu, Shanxi, China

**Keywords:** perilla seeds, desiccation tolerance (DT), ABA-GA antagonism, transcriptomic analysis, WGCNA

## Abstract

**Introduction:**

Perilla (*Perilla frutescens* (L.) Britt.), a valuable source of omega-3 oils and bioactive compounds in Asia, exhibits poor seed storage and germination performance. Understanding the genetic basis of desiccation tolerance (DT) during seed development is essential for improving perilla cultivation, yet these mechanisms remain largely unknown.

**Methods:**

We measured phenotypic and physiological parameters of perilla seeds at different developmental stages and performed transcriptome analysis to identify differentially expressed genes (DEGs). Using WGCNA, we correlated these DEGs with physiological traits to identify key modules.

**Results:**

We identified the D17-D27 stage as the critical window for DT acquisition in perilla seeds. Transcriptome analysis revealed 14,040 DEGs across different developmental stages. Through WGCNA analysis, we identified two key regulatory modules: the MEcoral module, which maintains membrane integrity through lipid metabolism, endoplasmic reticulum protein processing, and ABA signaling; and the MElavenderblush2 module, which regulates energy supply and cell wall remodeling via photosynthetic carbon metabolism and GA signaling. The core gene network (*ABI5/BBX22/MADS3*) suggests that the BBX family may serve as a crucial integrator, coordinating ABA, heat stress, and light signaling pathways to regulate antioxidant defense and energy metabolism, thereby enhancing seed adaptability.

**Discussion:**

This study elucidates the mechanisms underlying DT acquisition in perilla seeds and provides a theoretical basis for the genetic improvement of crop stress resistance.

## 1 Introduction

In the plant kingdom, desiccation tolerance (DT) is rare in buds and roots, but is more common in mature seeds and pollen (Leprince and Buitink, 2010). Seeds are the most important harvest organs in many crops, storing large amounts of essential substances used by humans and containing genetic material that can be used for crop improvement. Seed DT is gradually acquired during development (Wang et al., 2015). Seed development comprises two stages: embryo development and maturation (Park et al., 2013). During the embryo development stage, seeds acquire their basic structure and pattern through cell division and differentiation, and begin to synthesize dehydration-protective molecules; they then enter the maturation stage, which can be further divided into early and late maturation. During early maturation, seeds begin to acquire DT and accumulate large amounts of storage substances, including storage proteins, oils, and carbohydrates (Leprince et al., 2000).

From a physiological perspective, seeds that enter the maturation stage have already acquired both the ability to germinate and DT. During dehydration, longevity is gradually acquired (Leprince et al., 2017). Additionally, as seeds acquire DT, metabolic activity gradually decreases, thereby minimizing ROS production (Pammenter and Berjak, 2014). Excessive ROS production and limited antioxidant defense activity can induce oxidative stress. To alleviate this, many antioxidants, such as ascorbic acid, glutathione, and phenolic compounds, are believed to play a role (Kranner and Birtic, 2005). However, to effectively limit ROS production, photosynthesis must be inhibited (Farrant, 2000); during seed development, photosynthesis is typically suppressed in the maturation process. Chlorophyll degradation is impaired in seed maturation mutants, especially in severe alleles of *ABI3*, where chlorophyll degradation does not occur, and mature seeds retain green cotyledons (Delmas et al., 2013; Nambara et al., 1995).

The physiological ability to survive dehydration is acquired during development, typically coinciding with the stage of storage substance accumulation (Le et al., 2010; Verdier et al., 2013), and the accumulation of soluble carbohydrates is directly related to this process (Brenac et al., 1997). During dehydration, specific sugars form hydrogen bonds by replacing water molecules (Hoekstra et al., 2001), thereby preventing the harmful effects of dehydration on cell membranes. They also interact with proteins to prevent conformational changes that could lead to loss of protein function (Leprince et al., 1993). Desiccation-tolerant tissues typically contain high concentrations of sucrose and oligosaccharides, while the levels of reducing monosaccharides (such as fructose and glucose) are either very low or absent (Angelovici et al., 2010; Kuo et al., 1997). Abscisic acid (ABA) regulates multiple key processes during seed maturation, including the biosynthesis and transport of nutrients, chlorophyll degradation, tissue dehydration, and dormancy initiation (Bewley et al., 2013). In the early stages of embryo formation, the developing embryo is regulated by ABA from maternal tissues; in later stages, the hormone is synthesized by the seed itself (Graeber et al., 2012; Nambara and Marion-Poll, 2005; Finkelstein, 2013). During embryo formation, ABA prevents seed abortion and promotes embryo growth. In contrast, during the final stages of embryogenesis, ABA levels rise and act as a gibberellin (GA) antagonist by inhibiting embryo growth (Nambara and Marion-Poll, 2005; Finkelstein, 2013). Another aspect of seed development during DT acquisition is the induction of HSP and sHSP genes. During maturation, *HSFA9* is regulated by the developmental regulator ABI3 (Kotak et al., 2007), and interacts with *ABI5* and *DOG1* (Dekkers et al., 2016). *HSFA9* is also a target of inhibition by Aux/IAA proteins (Carranco et al., 2010; Righetti et al., 2015).

Perilla (*Perilla frutescens* (L.) Britt.) is a crop with medicinal, spice, and functional food value, widely distributed throughout China and East Asia. However, climate change has negatively impacted the yield and quality of perilla seeds, necessitating further investigation into their DT mechanisms. This study provides the first comprehensive analysis of the mechanisms underlying DT acquisition in perilla seeds, including physiological responses, gene expression regulation, and transcriptomic changes. It fills a gap in research on drought resistance in perilla seeds and provides a theoretical basis for future drought-resistant perilla breeding.

Perilla, as a unique model system for DT research, faces specific dehydration stress challenges, with significant impacts on yield and quality, especially under climate change. Our study found that during the critical period of DT acquisition in perilla seeds, DT is achieved through coordinated morphological, physiological, and genetic changes, specifically manifested as increased seed vigor, seed coat thickening, endosperm degradation, and reduced water content. Compared to previous research on seed development in Arabidopsis, soybean, and wheat (Rangan et al., 2017; Verma et al., 2022; Sun et al., 2020), this study employed more advanced multi-omics approaches, combining weighted gene co-expression network analysis (WGCNA) with physiological profiling to reveal gene modules associated with ABA signaling, lipid metabolism, and GA signaling pathways. The study also identified a novel role for the BBX gene family in regulating drought resistance. This integrated approach provides a more systematic and comprehensive perspective for understanding DT acquisition.

## 2 Materials and methods

### 2.1 Plant materials and experimental design

In late April 2023, the perilla variety Zisu No. 1 was planted at the Shanxi Agricultural University Entrepreneurship Park in Taigu District, Shanxi Province (Latitude: 37°25′38.2″N, Longitude: 112°32′40.5″E); seeds were donated by the North University of China. Climate data are provided in **Supplementary Table S1**. Starting in August, flowering spikes were marked daily to record flowering time. Sampling was conducted every 5 days beginning on day 7 (D7) after flowering, continuing until seed maturation. To clearly characterize physiological traits at different developmental stages, and based on previous research (Liao et al., 2018), the following sampling criteria were established: At the first sampling point (D7), seeds were in early embryogenesis, showing small size and a transparent to translucent appearance. At the second sampling point (D12), seeds showed increased volume with a color change from transparent to milky-white, along with more visible embryonic structures. The third sampling point (D17) represented the mid-developmental phase, with marked storage reserve accumulation and increased firmness, and distinct seed coat patterns. At the fourth sampling point (D22), seeds entered late development, showing progressive seed coat hardening. The fifth sampling point (D27) captured seeds approaching physiological maturity, with notable surface hardening and darker pigmentation. The final sampling point (D32) represented seeds at full physiological maturity, with completely hardened and dried seed coats.

Based on these sampling criteria, six temporal sampling points (D7, D12, D17, D22, D27, and D32 post-anthesis) were established, with samples taken from the middle portion of the flower spike (**Supplementary Figure S1**). Some seeds at different developmental stages were stored in a -80°C ultra-low temperature freezer for physiological measurements and transcriptome sequencing. Another portion was air-dried naturally in the laboratory for seed viability testing.

### 2.2 Observation of seed morphology and tissue structure

Ten perilla seeds were collected at 7, 12, 17, 22, 27, and 32 days after flowering. Intact fruits and dehusked seeds were observed and photographed under a Zeiss stereomicroscope (Stemi-2000-C, Germany). The method for observing cellular and tissue structures has been described in previous studies (Chen et al., 2024). Samples were stained with 0.1% (w/v) toluidine blue (Beijing Coolaber Science & Technology Co., Ltd) and incubated at 70-80°C for approximately 10–30 seconds. After rinsing off excess stain with distilled water, the samples were immediately observed and photographed under an OLYMPUS fluorescence microscope (BX53F, Japan).

### 2.3 Determination of agronomic traits and seed vigor

Perilla seeds were collected at 7, 12, 17, 22, 27, and 32 days after flowering. For each time point, 1000 fresh or dry seeds were randomly selected and weighed in triplicate, and mean values were calculated. The thousand-seed weight of dry perilla seeds was adjusted based on their actual moisture content to the standard moisture level (13%). The water content (WC) of seeds at different developmental stages was determined using the constant temperature oven method, following the guidelines of the International Seed Testing Association (ISTA) (Association, I. S, 2022). Eight grams of seeds were divided into four groups, with 2 g placed in each sealed weighing bottle (W1). Lids were removed and bottles were placed in the oven at 105°C for 5 hours, then the temperature was adjusted to 80°C to achieve constant weight. Samples were cooled in a vacuum jar containing anhydrous silica gel, lids were returned to the bottles, and the bottles were weighed (W2). Seeds were removed, and the empty container weight was recorded (W3). Water content is expressed as g H_2_O/g DW, calculated as follows:


$$\text{Water content}(\text{WC})=\frac{\left(\text{W}1-\text{W}2\right)}{\left(\text{W}2-\text{W}3\right)}$$

Germination tests were conducted according to the GB/5520-2011 “Germination Test for Grain and Oilseeds” (China, N. s. o. t. P. s. R. o, 2011). Since perilla seeds exhibit dormancy, we broke dormancy by treating them with a 200 mg/L GA_3_ solution. Each replicate consisted of 200 seeds, with four replicates in total. The seeds were placed in Petri dishes (approximately 15 cm in diameter) containing two layers of filter paper, to which 5 mL of ddH_2_O was added. The seeds were incubated at 25°C under a 14/8-hour light/dark photoperiod for 7 days. Seeds were considered germinated when the radicle emerged from the seed coat. After the germination test, the remaining husks were removed, and the seeds were blanched at 105 °C for 30 minutes, then dried at 80°C for 24 hours. The dry weight of individual seedlings was then measured. The calculation formulas are as follows:

$\text{Germinating energy }(\text{GE})=\frac{\text{Germination number of seeds within }3\text{ days}}{\text{Number of test seeds}}*100\%$

$\text{Rate of germination }(\text{GR})=\frac{\text{Germination number of seeds within }7\text{ days}}{\text{Number of test seeds}}*100\%$


$$\text{Germination index }(\text{GI})=\sum \frac{\text{Gt}}{\text{Dt}}$$

In the formula, Gt represents the number of seeds germinated on the corresponding day Dt, where Dt is the germination day.


Vital index (VI)=GI*S

In the formula, GI represents the germination index, and S is the total number of seedlings’ dry weight (g).

### 2.4 Measurement of physiological indicators

According to the sampling method, fresh seeds at different developmental stages were placed in liquid nitrogen and ground into a fine powder. Three replicates were performed for each sampling period, which were used for the following physiological measurements. Following the instructions provided by the corresponding reagent kit (Beijing Boxbio Science & Technology Co., Ltd.), for each replicate, 0.1 g of the powder was used to measure malondialdehyde (MDA), hydrogen peroxide (H_2_O_2_), superoxide anion (O_2_^−^), catalase (CAT), superoxide dismutase (SOD), and peroxidase (POD) activity. Using 0.15 g of the powder, the content of soluble total sugars (SS), sucrose (Suc), fructose (Fru), and glucose (Glu) was measured. Abscisic acid (ABA) and gibberellin (GA) were detected using a plant ELISA detection kit (Enzyme Biotechnology Co., Ltd., China). The absorbance of the reaction mixture was measured using a Molecular Devices microplate reader (USA).

### 2.5 Transcriptome sequencing and analysis

Seeds were collected at 7, 12, 17, 22, 27, and 32 days after pollination during perilla development, with three biological replicates for each developmental stage. The transcriptome sequencing process, including total RNA extraction, mRNA purification, cDNA synthesis, and sequencing, was performed by Nanjing Paisenno Biotechnology Co., Ltd. (Nanjing, China). The reference genome and analysis methods were as described in previous studies (Chen et al., 2024). To improve the reliability of the results, perilla protein sequences were aligned against the SwissProt plant protein database using BLAST, and the “GN=“ field was extracted from the alignment results for gene name annotation. High-similarity alignment results were used to annotate the perilla genes, while maintaining their correspondence with genes from the model plant *Arabidopsis*. GO and KEGG enrichment analyses were conducted using the plant databases.

### 2.6 Weighted gene co-expression network

To identify co-expression modules and key regulatory genes associated with target traits in perilla seeds, we used the WGCNA package in R for analysis, as described in previous studies on co-expression networks (Langfelder and Horvath, 2008). Briefly, only genes with an average FPKM value greater than 0.5 were retained, and a log2(FPKM + 1) transformation was applied before further processing. The pickSoftThreshold function based on the scale-free topology model was used to determine the soft-thresholding power with R^²^ > 0.8. Subsequently, the automatic blockwiseModules construction method was applied to obtain highly correlated modules, with the following parameters: power = 22, TOM-type = unsigned, minimum module size = 50, maximum block size = 35,000, merge cut height = 0.25.

### 2.7 Screening and functional analysis of core genes

To further explore the functions of genes in the important modules, GO and KEGG functional enrichment analyses were performed on the genes within these modules, with a threshold of P < 0.05. After multiple testing correction, the entries that met this condition were considered significantly enriched. Based on the iTAK (iTAK - Plant Transcription factor & Protein Kinase Identifier and Classifier) alignment results using Arabidopsis as a model plant, transcription factors (TFs), transcription regulators (TRs), and receptor kinases (RKs) with higher connectivity were identified. These genes were considered core genes within the module and were used to construct a co-expression network. Functional analysis of the core genes was conducted based on existing research findings. The co-expression regulatory network was visualized using Cytoscape v3.5.1.

### 2.8 qRT-PCR validation

Based on the CDS sequences obtained from RNA-seq, primers were designed using the Primer-BLAST tool on the NCBI website. The primers were synthesized by Shenggong Biotech (Shanghai) Co., Ltd. (**Supplementary Table S2**). A qRT-PCR kit produced by Enzymes Biotech Co., Ltd (Jiangsu, China) was used, and the reaction system is shown in **Supplementary Table S3**. *Actin* and *18S RNA* were used as reference genes, and the data were analyzed using the 2-ΔΔCt method.

### 2.9 Statistical analysis

Data were analysis using Excel 2022 (Microsoft, Redmond, WA, USA) and SPSS 23.0 software (SPSS Inc., Chicago, IL, USA). We performed one-way analysis of variance (ANOVA) and applied Duncan’s multiple comparison method to determine significant differences between groups.

## 3 Results

### 3.1 Agronomic traits and seed vigor

**Figure 1:**
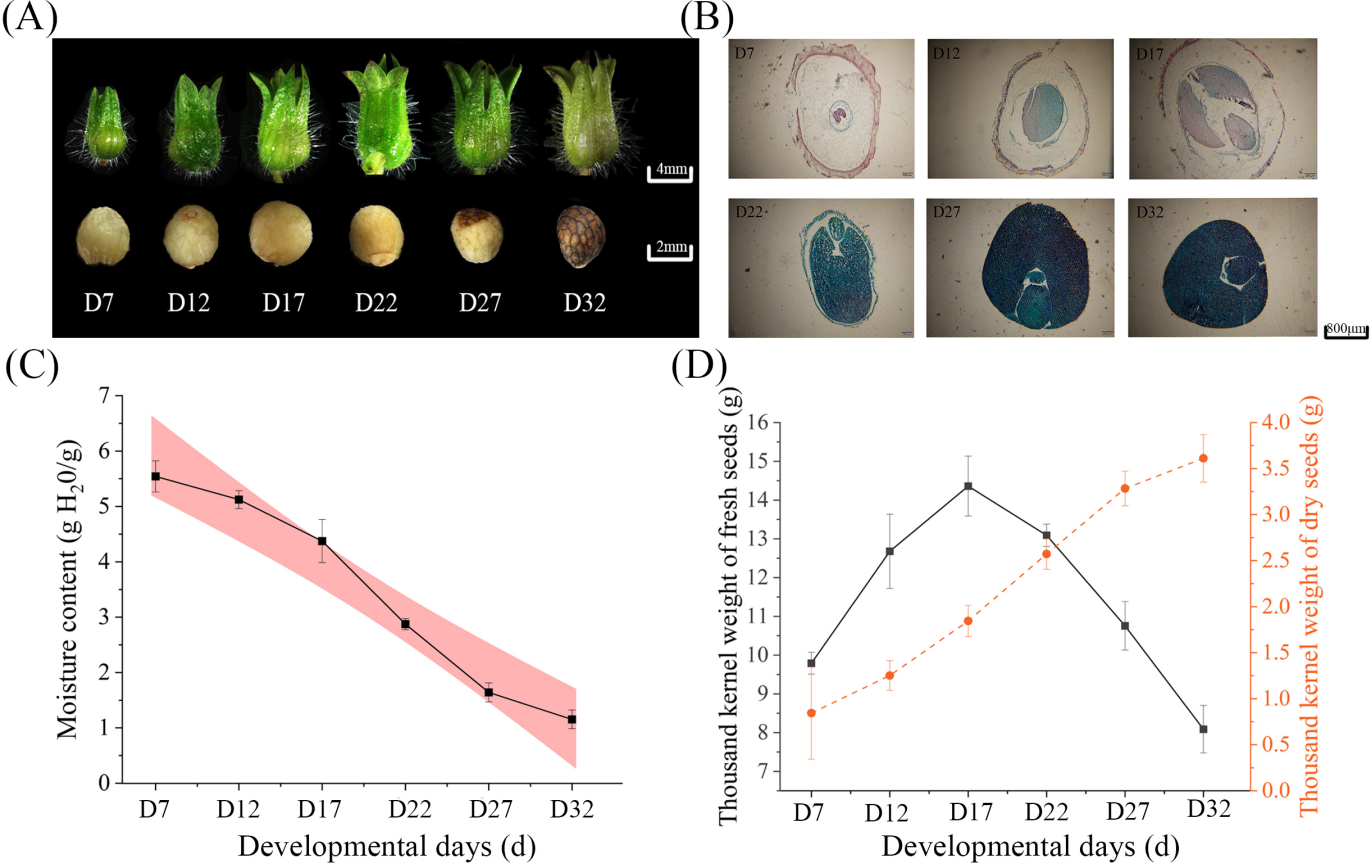
**(A)** Morphological images of seeds at different developmental stages (D7, D12, D17, D22, D27, D32), showing changes in seed size and structure. **(B)** Histological cross-sections of seeds at different stages, stained to visualize internal structural changes during development. **(C)** The trend of moisture content in seeds during development. **(D)** The thousand-seed weight of fresh seeds (black line) and dry seeds (orange line) at different developmental stages.

This study characterized the dynamic changes in perilla seeds during DT acquisition and morphological development. Using stereoscopic and fluorescence microscopy, we documented the morphological development and internal tissue structure changes of perilla seeds from 7 to 32 days after pollination (**Figures 1A, B**).

Between D7 and D17, seed moisture content gradually declined, coinciding with cell expansion and storage substance accumulation. From D17 to D27, seeds underwent rapid dehydration with a sharp reduction in moisture content. Between D27 and D32, the dehydration rate slowed (**Figure 1C**), indicating the establishment of mature dehydration protection mechanisms. Analysis of 1000-seed weight dynamics (**Figure 1D**) revealed that fresh weight peaked at D22 before declining significantly, while dry weight continued accumulating until stabilizing at D27. These results suggest that the period between D22 and D27 represents a critical window for the acquisition of DT.

During the development of perilla seeds, various vigor indicators exhibited significant dynamic changes as the number of days after pollination increased (**Table 1**). From day 7 to day 12 after pollination, the seeds were in the early stage of embryo development, with germination rate, germination potential, vigor index, and germination index all close to zero. Starting from day 17 after pollination, the seeds entered the phase of acquiring DT, and the germination rate, vigor index, and germination index increased significantly. Day 27 after pollination marked the peak vitality period of the seeds, with a germination rate of 95.75% ± 0.25%, vigor index (5.91 ± 0.20), and germination index (17.42 ± 0.08) all reaching their maximum values. This indicated that at this point, the seeds had completed the establishment of their dehydration protection mechanisms and possessed optimal field germination potential. Notably, although the germination potential of the seeds increased to 47.75% ± 1.37% on day 32 after pollination (significantly higher than the 40.00% ± 2.19% on day 27, p < 0.05), their germination rate and vigor index decreased to 88.75% ± 0.62% and 5.29 ± 0.17, respectively.

**Table 1:** Seed Viability Determination.

Developmental days (d)	Germination rate (%)	Germinating energy (%)	Vitality index	Germination index
D7	0 ± 0.00^e^	0 ± 0.00^e^	0 ± 0.00^d^	0 ± 0.00^e^
D12	1.75 ± 0.47^e^	0 ± 0.00^e^	0.02 ± 0.02^d^	0.20 ± 0.02^e^
D17	52.00 ± 1.95^d^	4.75 ± 1.25^d^	0.95 ± 0.08^c^	6.60 ± 0.24^d^
D22	77.00 ± 1.58^c^	20.50 ± 1.93^c^	2.79 ± 0.02^b^	12.39 ± 0.30^c^
D27	95.75 ± 0.25^a^	40.00 ± 2.19^b^	5.91 ± 0.20^a^	17.42 ± 0.08^a^
D32	88.75 ± 0.62^b^	47.75 ± 1.37^a^	5.29 ± 0.17^a^	16.24 ± 0.26^b^

Different letters mean significant difference among treatments (P < 0.05).

The above results suggest that the acquisition of DT in perilla seeds is a staged regulatory process: morphological development (D7-D17) provides the structural foundation, physiological maturation (D17-D22) activates protective mechanisms, and mature dehydration (D22-D32) completes the adaptive transition. Specifically, the critical window from day 17 to day 27 after pollination, during which the seeds gradually acquire DT, provides important evidence for determining the optimal harvesting time of perilla seeds (27 days after pollination).

### 3.2 Changes in physiological characteristics during the DT acquisition process

**Figure 2:**
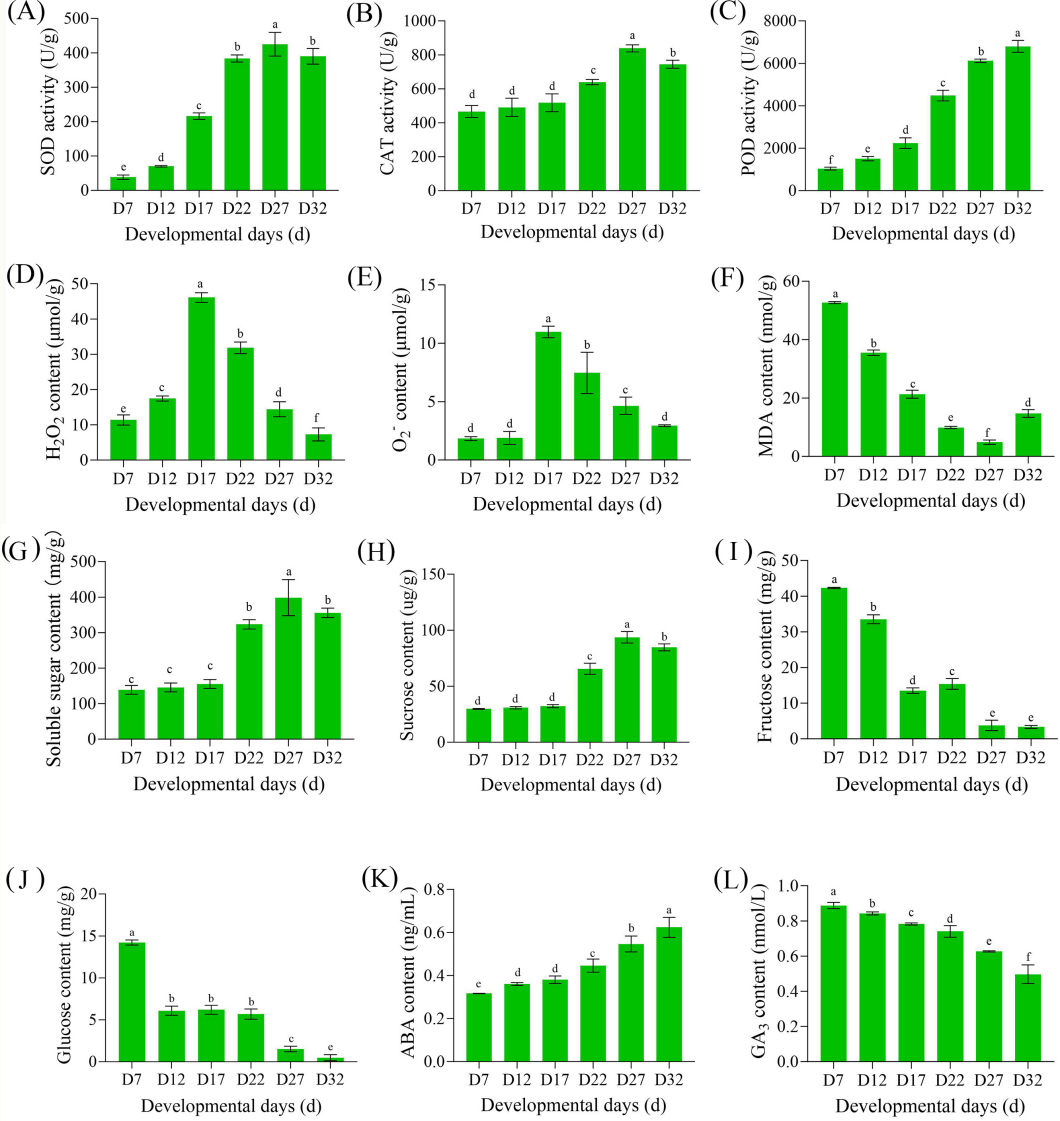
Changes in antioxidant enzyme activities, reactive oxygen species levels, sugar contents, and other physiological parameters during seed development. **(A)** Peroxidase (POD) activity, **(B)** Catalase (CAT) activity, **(C)** Superoxide dismutase (SOD) activity, **(D)** Hydrogen peroxide (H_2_O_2_) content, **(E)** Superoxide anion (O_2_^−^) content, **(F)** Malondialdehyde (MDA) content, **(G)** Soluble sugar content, **(H)** Sucrose content, **(I)** Fructose content, **(J)** Glucose content, **(K)** abscisic acid (ABA) content, and **(L)** Gibberellin (GA) content at different developmental stages (D7, D12, D17, D22, D27, D32) in perilla seeds. Values are means ± SE (n = 3). Different letters above bars indicate significant differences among developmental stages (P < 0.05).

During the acquisition of DT in plants, changes in reactive oxygen species (ROS) and the antioxidant system are key factors (Laxa et al., 2019), while the relationship between sugars and hormones mainly involves sugar accumulation, hormone signaling, and the regulation of enzymatic systems (Bolouri-Moghaddam et al., 2010; Nobiza et al., 2023). To clarify the physiological regulatory mechanisms of DT acquisition in perilla seeds, we measured the levels of these indicators. In the antioxidant enzyme system, SOD, CAT, and POD showed an increasing trend with seed development (**Figures 2A–C**), while ROS levels (H_2_O_2_ and O_2_^-^) first increased and then decreased (**Figures 2D, E**), indicating that the antioxidant system mitigates dehydration-induced damage during development. The membrane lipid peroxidation product (MDA) further confirmed this, showing a decreasing trend during the DT acquisition process (**Figure 2F**). As seed development progressed, soluble sugar content remained constant from D7 to D17, increased to the highest level at D27, and then decreased (**Figure 2G**). The trend in sucrose content was consistent with that of soluble sugars (**Figure 2K**), indicating that sucrose was the primary form of sugar accumulation. Fructose and glucose levels were high in the early stages of development (D7) and decreased to their lowest levels as DT was fully acquired (**Figures 2M, N**). Notably, the change in ABA content aligned with the DT acquisition process during seed development, with the greatest increase in ABA content occurring between D27 and D32 (**Figure 2O**). Conversely, GA content showed the opposite trend to ABA content (**Figure 2P**), indicating that these two major hormones exhibited an antagonistic relationship during the DT acquisition process in seeds.

### 3.3 DT obtains the function of process DEGs

To further elucidate the gene regulatory mechanisms during the development of perilla seeds, this study conducted transcriptome sequencing on samples from various stages (D7, D12, D17, D22, D27, and D32), with three biological replicates at each stage. The mapping rate exceeded 97% (**Supplementary Table S4**), indicating that the experimental data were reliable and had good reproducibility. Additionally, gene expression curves at different time points showed subtle differences, reflecting the dynamic changes in gene expression (**Supplementary Figure S2A**). The first principal component (PC1) explained most of the variance, demonstrating biological reproducibility and significant differences in gene expression patterns across different developmental stages (**Supplementary Figure S2B**).

**Figure 3:**
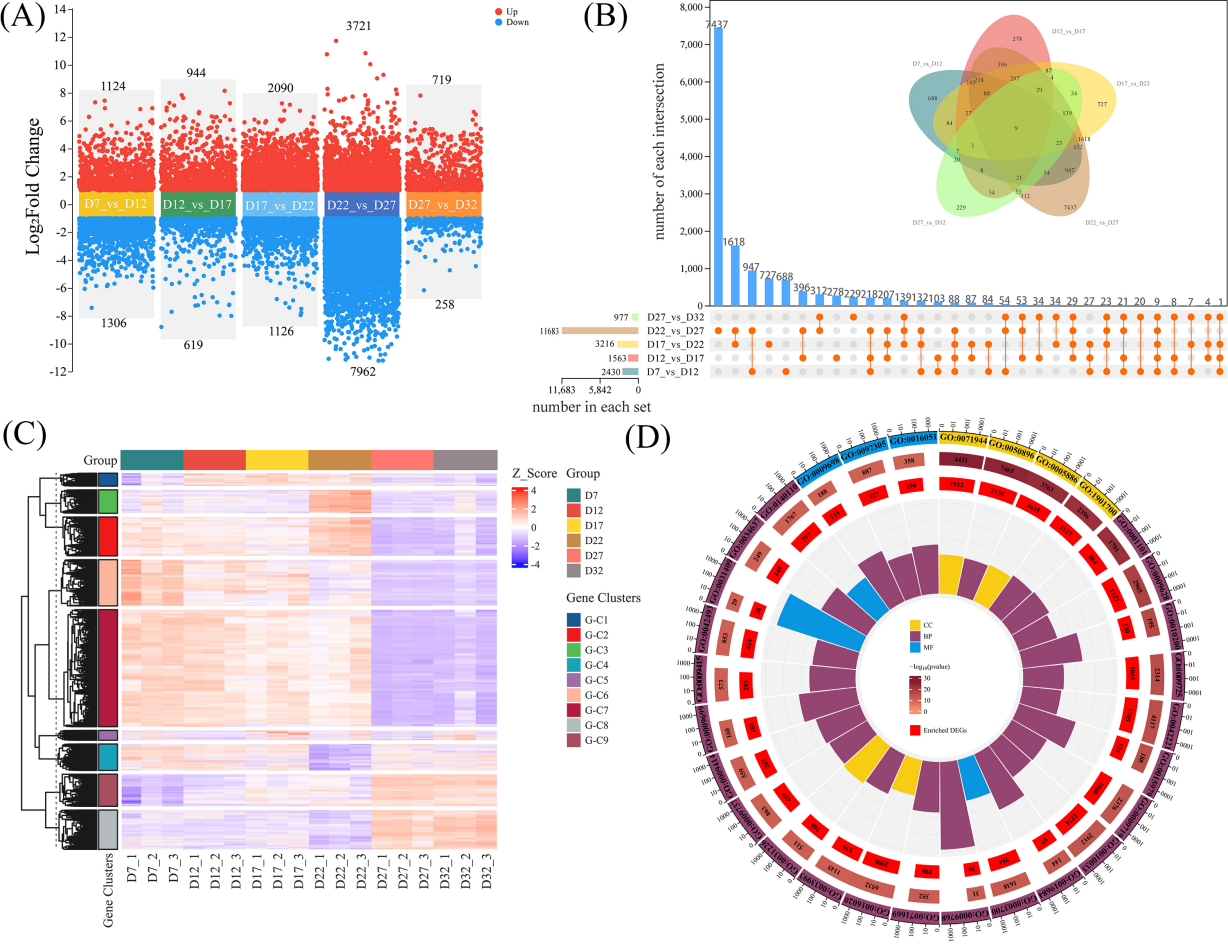
**(A)** Comparison of DEGs between adjacent stages of perilla seed development. Red and blue dots represent upregulated and downregulated genes, respectively. **(B)** Venn diagram and bar plot showing the union of DEGs across all comparison groups. Each intersection represents the number of common genes between the groups, with the total number of DEGs in each comparison listed below. **(C)** Heatmap analysis of the union DEGs, where the Z-score represents the expression level. The DEGs are divided into different gene clusters and labeled with corresponding tags. **(D)** Gene Ontology (GO) enrichment analysis of the union DEGs, displayed in a circular plot. The plot highlights enriched GO terms and categorizes genes based on their functions, including cellular components (CC), biological processes (BP), and molecular functions (MF).

The volcano plot (**Figure 3A**) displays the results of pairwise comparisons across different developmental stages, revealing significant differences in gene expression between stages. Subsequently, we performed a differential expression analysis using the union of all comparison groups throughout the developmental process. The results showed a total of 14,040 DEGs, with genes exhibiting significant expression changes in at least one developmental stage or comparison group (**Figure 3B**). In the clustering heatmap, these DEGs exhibited nine distinct expression patterns (**Figure 3C**). We observed that G-C2, G-C6, and G-C7 shared the same expression pattern during the D27-D32 stage. According to the GO enrichment results (**Figure 3D**; **Supplementary Table S5**), many terms were related to hormone signaling (“response to hormone” GO:0009725, especially “response to abscisic acid” GO:0009737), carbohydrate metabolism (“carbohydrate biosynthetic process” GO:0016051), and environmental or stress responses (“response to abiotic stimulus” GO:0009628, “response to water deprivation” GO:0009414, etc.). KEGG pathway analysis also showed significant enrichment in “Plant hormone signal transduction” (ko04075) and various carbohydrate metabolism-related pathways (e.g., “Starch and sucrose metabolism” ko00500, “Galactose metabolism” ko00052, “Glycolysis” ko00010, etc.) (**Supplementary Figure S3**; **Supplementary Table S6**). These processes include hormone regulation, carbohydrate metabolism, cell protection, water balance, and cell wall remodeling.

### 3.4 Functional genes responsive to DT during the developmental process

**Figure 4:**
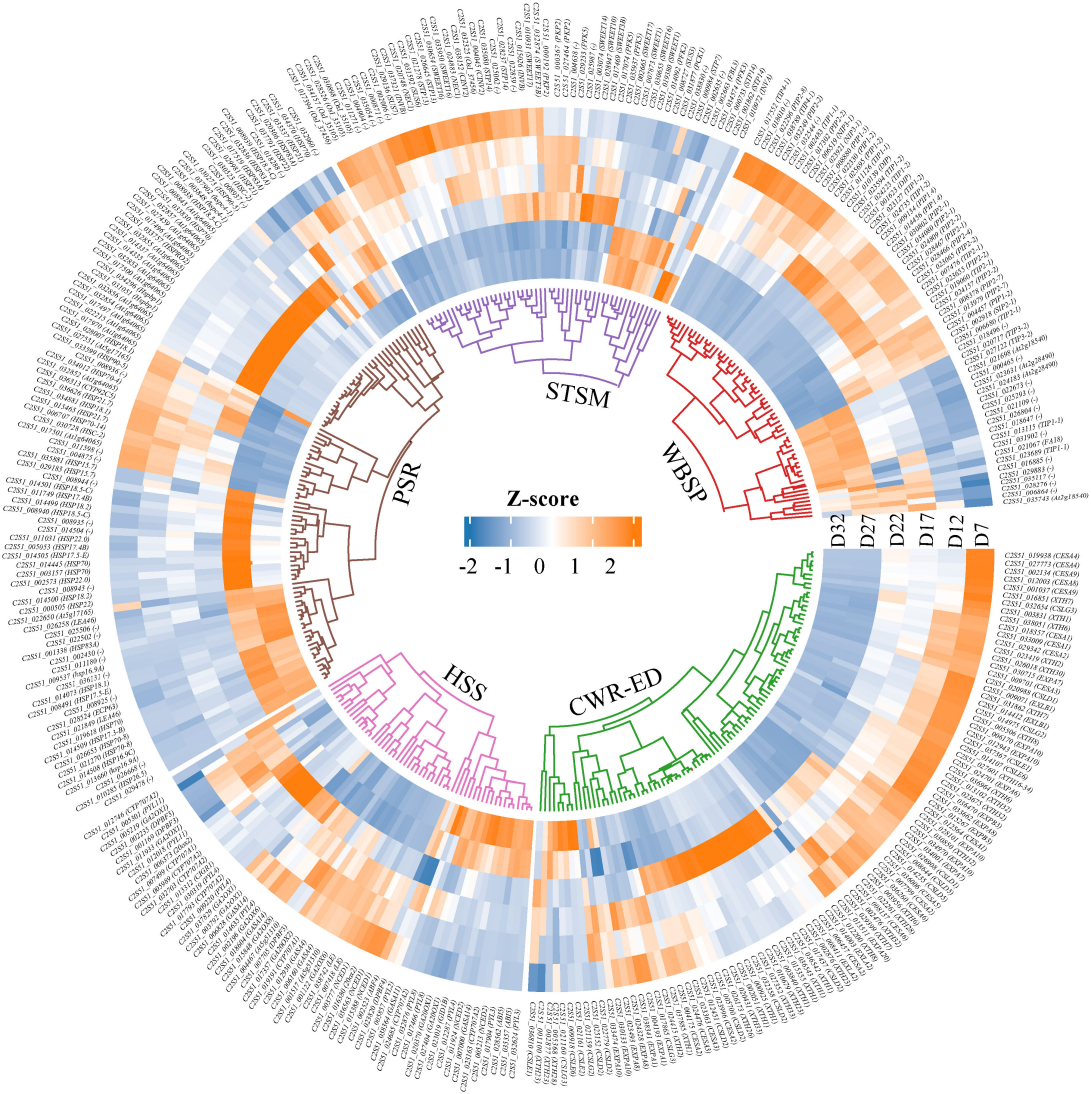
The clustering heatmap of DEGs in five functional categories. The specific classifications are as follows: Hormone Synthesis and Signaling (HSS) includes 9-cis-epoxycarotenoid dioxygenase (NCED), Abscisic Acid (ABA), and Gibberellin (GA). Sugar Transport and Sugar Metabolism (STSM) includes Sugar Transport Protein (STP), Sucrose Synthase (SUS), Invertase (INV), Phosphofructokinase (PFK), and Pyruvate Kinase (PK). Protective/Stress-Resistant Proteins (PSR) includes Late Embryogenesis Abundant Protein (LEA) and Heat Shock Protein (HSPs). Water Balance and Storage Proteins (WBSP) includes Aquaporins (AQP) and Seed Storage Proteins (SSP). Cell Wall Remodeling and Embryo Development (CWR-ED) includes Expansin (EXP), Xyloglucan Endo-Transglucosylase Protein (XTH), and Cellulose Synthase (CES). The Z-score value represents the gene expression level, with red indicating high expression, and blue indicating low expression.

Clustering analysis and GO/KEGG functional enrichment analysis of DEGs across all time points revealed that genes related to seed DT undergo dynamic changes across multiple physiological processes. Bused on these results, we further classified the DEGs closely associated with DT into five major functional gene categories: Water Balance and Storage Proteins (WBSP), Hormone Synthesis and Signaling (HSS), Sugar Transport and Sugar Metabolism (STSM), Cell Wall Remodeling and Embryo Development (CWR-ED), and Protective/Stress-Resistant Proteins (PSR) (**Figure 4**; **Supplementary Table S7**). The heatmap results for these functional categories show that the genes in the WBSP, HSS, and STSM categories primarily exhibit two expression patterns: one where expression increases during early seed development and is then downregulated; and another where genes are significantly upregulated during the D27–D32 stage but suppressed during the D7–D17 stage. Most CWR-ED and PSR genes also follow these patterns; notably, a subset of these genes is significantly upregulated at D17 while being suppressed at other stages. These findings indicate that gene expression within these functional categories is precisely regulated at different stages of seed development to meet specific physiological demands and environmental conditions, thereby playing key roles in the acquisition of DT.

### 3.5 TFs, TRs, and PKs associated with DT acquisition

**Figure 5:**
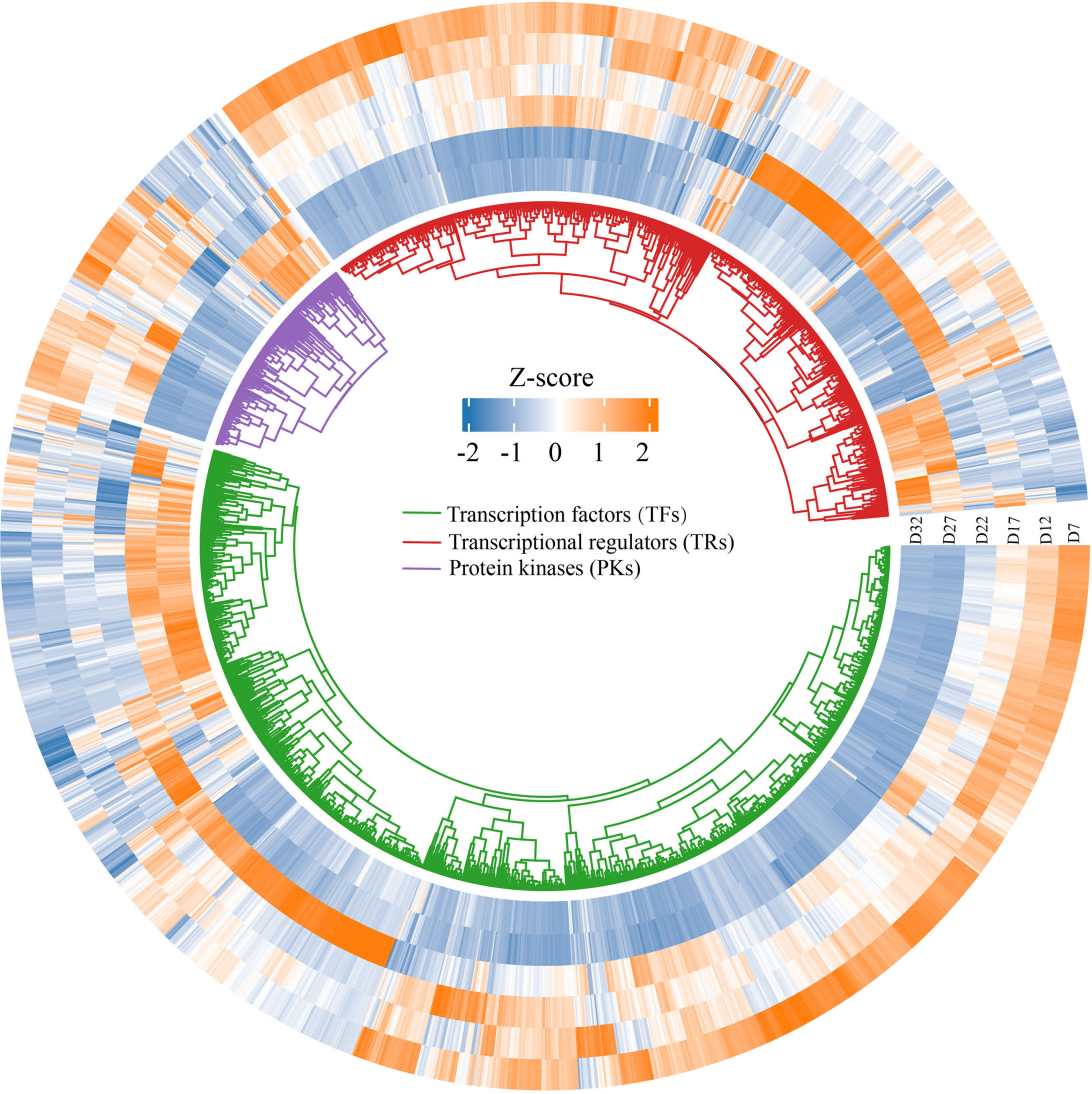
Circular heatmap displaying the expression patterns of differentially expressed genes across various developmental days (D7, D12, D17, D22, D27, D32). The heatmap is organized by three gene categories: Transcription factors (TFs) (green), Transcriptional regulators (TRs) (blue), and Protein kinases (PKs) (purple).

During seed development, transcription factors (TFs), transcriptional regulators (TRs), and protein kinases (PKs) dynamically cooperate to regulate key stages such as embryogenesis, storage substance accumulation, and desiccation maturation (Lepiniec et al., 2018; Weinman et al., 2018). We screened 1,134 TFs, 205 TRs, and 701 PKs that were further screened from the 14,040 DEGs using the iTAK plant online tool (**Supplementary Table S8**), and found that the TFs were mainly classified into families such as MADS, MYB, NAC, bZIP, and WRKY. Their expression patterns varied across different stages of seed development (**Figure 5**). The heatmap shows that the expression profiles of TFs, TRs, and PKs can be categorized into three groups. The first group is significantly upregulated at D7 and subsequently downregulated during the D27–D32 stage. The second group is strongly upregulated at D22, while being suppressed or not expressed at other stages. The third group shows significant upregulation during the D27–D32 stage. Overall, these three groups of genes exhibit diverse expression patterns along the developmental timeline, reflecting the dynamic regulatory features involved in the formation of DT, from early responses to late-stage adaptations.

### 3.6 WGCNA analysis

To analyze the molecular regulatory network of seed DT acquisition, it is crucial to select key traits that can comprehensively reflect seed DT and its developmental process using WGCNA. The moisture-related indicators MC and FSW reflect changes in the dehydration process and water status during seed development, while GR directly assesses the seed’s germination ability and vigor. Changes in antioxidant enzymes (SOD, CAT, POD), reactive oxygen species (H_2_O_2_, O_2_^–^), and the oxidative damage marker MDA reveal how seeds cope with oxidative stress through antioxidant defenses during dehydration. Furthermore, soluble sugars (SS, Suc, Fru, Glu), as energy sources and osmotic regulators, help maintain the stability of seed cell membranes. The regulatory roles of GA and ABA in seed development and maturation, through the modulation of growth and dormancy balance, have a significant impact on DT.

**Figure 6:**
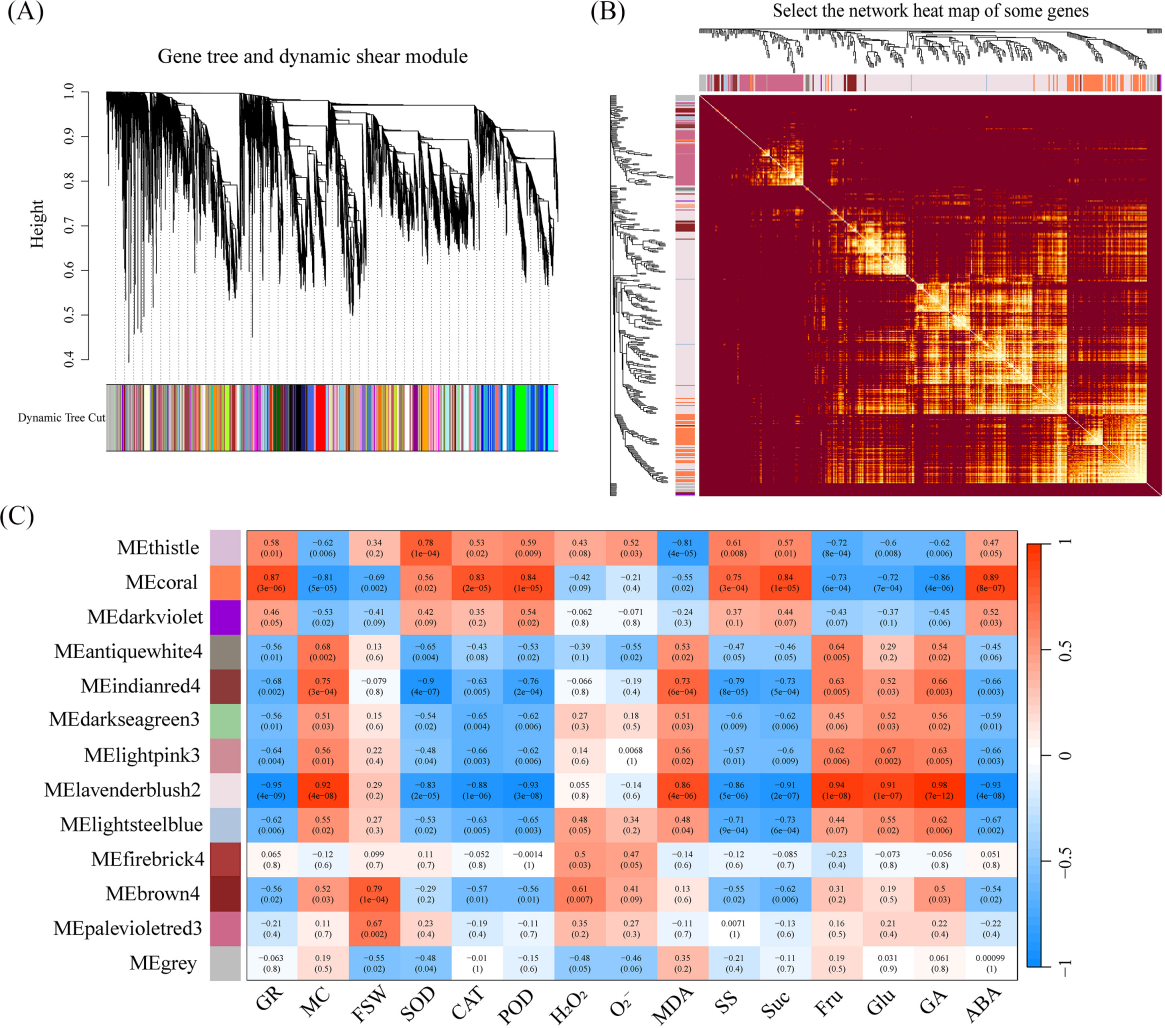
**(A)** The hierarchical clustering dendrogram shows the gene tree with the dynamic tree cut applied. Different colors in the bottom bar represent distinct gene modules identified by the dynamic tree cut method. **(B)** The gene network heatmap shows clustered genes, and the intensity of color reflects the strength of correlations between gene expressions, with brighter areas indicating stronger associations. **(C)** The module-trait correlation heatmap displays the correlation between each module and different traits. The color bar represents the strength of the correlation, with red indicating positive correlations and blue indicating negative correlations.

In crops, complex traits are often regulated by multiple transcriptional networks. To identify co-expression networks associated with target traits, we used WGCNA analysis and found that sample clustering and correlation coefficients showed strong reproducibility between biological replicates, eliminating the need to remove outliers (**Supplementary Figure S4A**). The optimal soft threshold was 22, with the fitting curve approaching 0.8 (**Supplementary Figure S4B**). Based on TOM (Topological Overlap Matrix) dissimilarity measurements, all candidate genes were clustered into different modules with similar expression profiles. Using the dynamic tree cutting method with minModuleSize = 30, 14,040 candidate genes were divided into 75 modules (**Figure 6A**). Similar modules were clustered by calculating the eigengenes and dissimilarity of the module eigengenes. After using the merge function, 1,000 genes were selected, and a network heatmap based on their TOM similarity matrix was plotted (**Figure 6B**), ultimately merging the original 75 modules into 10 modules (**Figure 6C**).

Our next goal was to identify modules and hub genes controlling these traits by combining trait characteristics with the expression trends of DEGs (**Figure 3C**). To achieve this goal, we used the Pearson correlation coefficient (p < 0.05) to determine which ME modules are significantly associated with traits. Interestingly, MEcoral was significantly positively correlated with the traits GR, CAT, POD, SS, Suc, and ABA. MElavenderblush2 was significantly positively correlated with the traits MC, MDA, Fru, Glu, and GA (**Figure 6C**). Furthermore, MEcoral and MElavenderblush2 were significantly negatively correlated with each other. Based on these correlations, we designated MEcoral as the Membrane Homeostasis Module and MElavenderblush2 as the Dehydration Response Module, as they were strongly associated with specific seed traits related to water status, oxidative stress response, and energy metabolism during seed development.

### 3.7 RT-qPCR verification results

**Figure 7:**
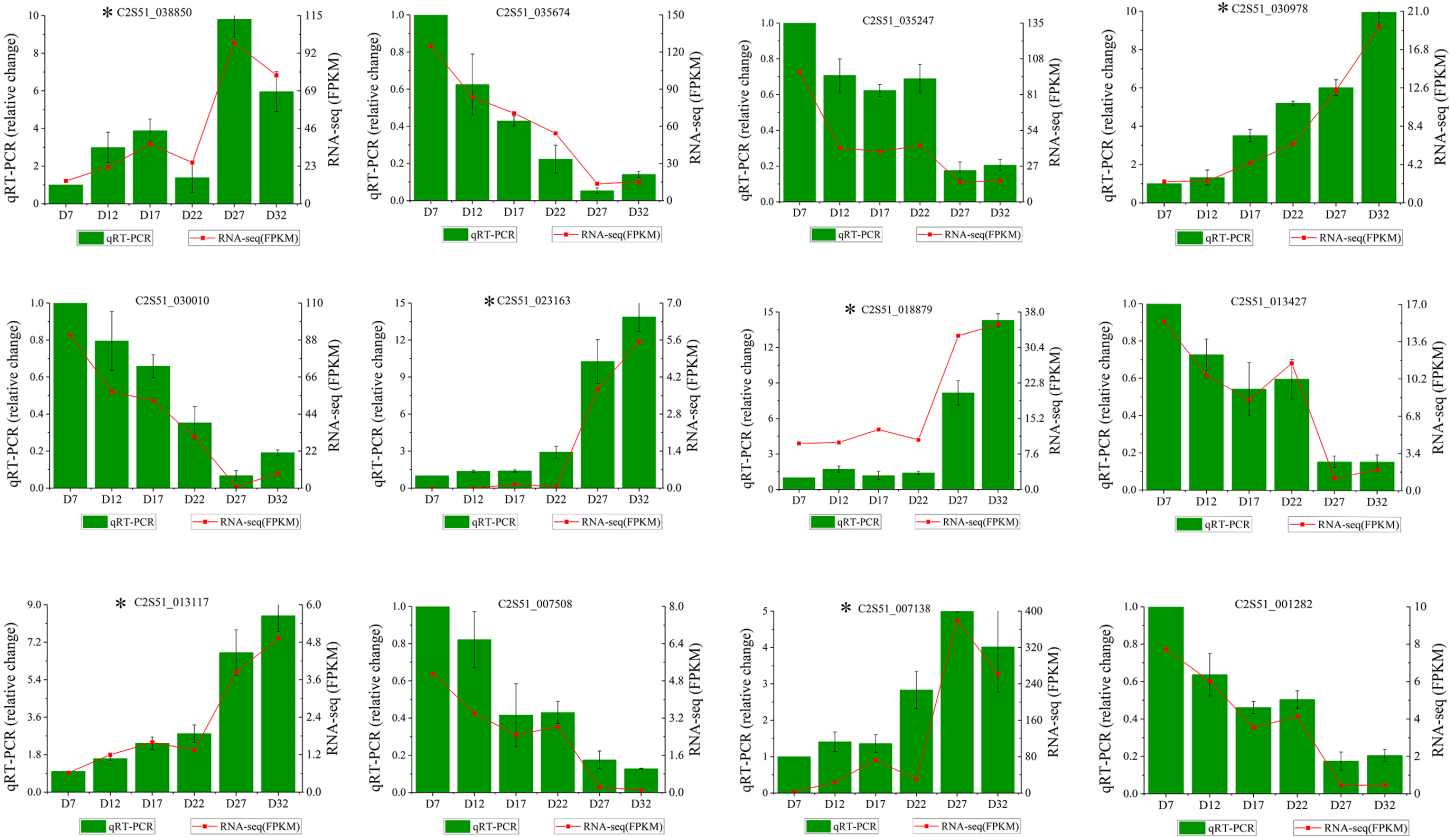
qRT-PCR verification results. The x-axis represents samples from different developmental stages, and the gene expression levels detected by qRT-PCR and RNA-seq are displayed on the left and right y-axes, respectively. The bars and red lines represent the results of qRT-PCR and RNA-seq, respectively. Genes marked with asterisks (*) in the figure are from the MEcoral module, while unmarked genes are from the MElavenderblush2 module.

To verify the accuracy of the RNA-seq results, we randomly selected key genes from the two modules mentioned above. Using *Actin* and *18S RNA* as reference genes, we calculated the expression levels of these 12 genes through qRT-PCR. The qRT-PCR results for the 12 selected genes were consistent with the FPKM value changes observed in the RNA-seq data (**Figure 7**). The validation results indicate that the transcriptome sequencing data are reliable and can be used for subsequent analyses.

### 3.8 GO and KEGG functional enrichment analysis

**Figure 8:**
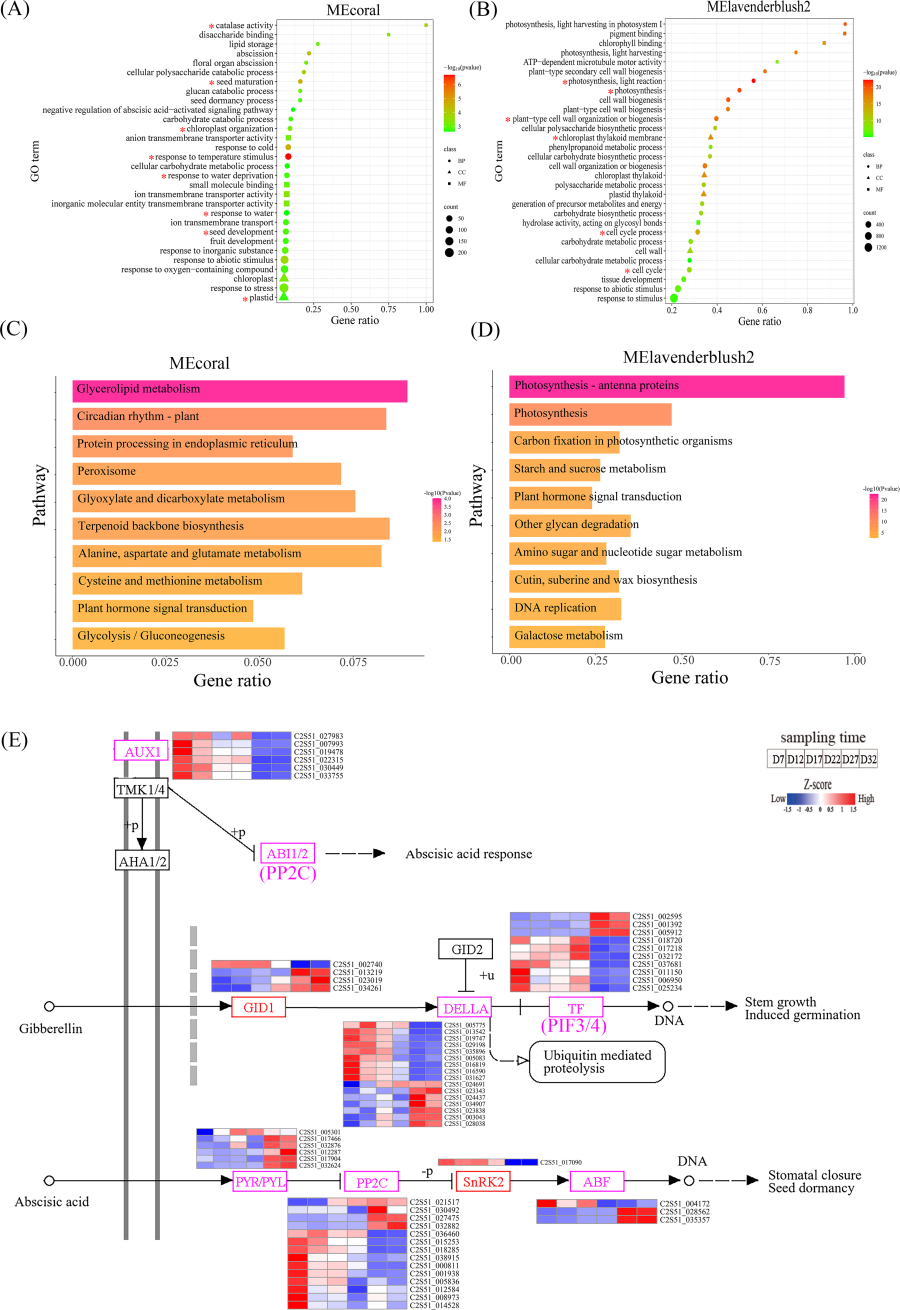
**(A, B)** show the GO analysis results for the MEcoral and MElavenderblush2 modules, displaying the relationship between gene ratio and significance. The items of interest in the module GO enrichment analysis are marked with an asterisk (*). The colors range from red to green, indicating different P-values, and the size of the circles represents the number of genes. **(C, D)** show the KEGG pathway enrichment analysis for the MEcoral and MElavenderblush2 modules, illustrating the relationship between different pathways and gene ratios. The colors range from red to yellow, indicating different P-values, and the pathways are sorted by gene ratio. **(E)** shows a detailed diagram of the ko04075 pathway, involving the signaling pathways of plant hormones GA and ABA. The color bar represents gene expression levels at different time points, from low to high (blue to red).

Based on these findings, we performed GO enrichment analysis on the genes of these two color modules. The MEcoral module contains 1,736 DEGs that are significantly enriched for processes of response to temperature stimulus, seed maturation, catalase activity, response to water deprivation, and chloroplast function (**Figure 8A**). These results indicate that the genes in this module play an important role in plant responses to environmental stresses, such as temperature and water deficiency. The MElavenderblush2 module contains 7,623 DEGs that are significantly enriched in processes such as photosynthesis, light reaction, chloroplast thylakoid membrane, plant-type cell wall organization or biogenesis, and cell cycle process (**Figure 8B**). This suggests that the genes in this module play a key role in important biological processes and structures, including photosynthesis, plant cell wall biosynthesis, cell cycle, and chloroplast function.

The KEGG enrichment analysis results show that the MEcoral module is primarily enriched in pathways such as glycerolipid metabolism, circadian rhythm-plant, and protein processing in the endoplasmic reticulum (**Figure 8C**). These pathways suggest that, the activation of peroxisomal metabolism pathways and the maintenance of cellular membrane homeostasis together constitute a key mechanism for DT. The MElavenderblush2 module is significantly enriched in photosynthesis-related pathways, including photosynthesis - antenna proteins, carbon fixation in photosynthetic organisms, and starch and sucrose metabolism (**Figure 8D**). Notably, this module coordinates the regulation of photosynthetic energy conversion, carbon assimilation efficiency, and sugar metabolism balance, providing the essential energetic foundation for seed stress resistance responses. Meanwhile, the ABA mediated by this module and GA_3_ from the MEcoral module jointly participate in a plant hormone signal transduction network, forming cross-regulatory mechanisms and establishing a multi-dimensional dehydration stress response system from energy supply to signal perception.

To clarify the expression patterns of DEGs in the plant hormone signaling pathway (Ko04075) during the DT acquisition process of perilla, we analyzed the 280 DEGs involved in the two modules and identified key genes related to the regulation of ABA and GA signaling pathways (**Figure 8E**). The results indicate that the soluble gibberellin receptor protein *GID1*, growth inhibitor protein *DELLA*, and transcription factors *PIF3/4* in the GA pathway exhibit two main expression patterns: one in which gene expression shifts from upregulation to downregulation during development, and another in which expression shifts from downregulation to upregulation. In contrast, the core components of the ABA pathway, including ABA receptors *PYR/PYL*, protein phosphatase *PP2C*, protein kinase *SnRK2*, and transcription factors *ABF/ABI5*, exhibit the same two expression patterns as those found in the GA pathway. In the auxin signaling pathway coordinated with ABA and GA, *AUX1* (*LAX2/3*) transporters are highly expressed during early development (D7-D12) and their expression is significantly suppressed later in development. Furthermore, *TMK1/4* may regulate *ABI1/2* activity through phosphorylation, thereby influencing the response of the ABA signaling pathway.

### 3.9 Hub genes and their co-expression networks

To identify the hub genes in the modules of interest, we first evaluated the absolute value of the Pearson correlation for gene connectivity (Kwithin). Then, we used the top 30% of genes by Kwithin as the hub genes for these modules (Niu et al., 2020). The K values for the MEcoral module genes range from 121.4 to 305.9. According to the iTAK analysis results, this module successfully identified 37 TFs, 8 TRs, and 18 PK genes. The hub genes in the module with high Kwithin include the TF gene C2S51_035357 (*ABI5*, K = 300.3), the TR gene C2S51_036594 (*BBX22*, K = 266.4), and the PK gene C2S51_018879 (*fray2*, K = 297.5), which were selected as the central genes of the MEcoral module. For the MElavenderblush2 module, we identified 233 TFs, 26 TRs, and 116 PKs among the hub genes. The K values range from 249.4 to 668.3. The hub genes in this module with high Kwithin include the TF gene C2S51_025892 (*MADS3*, K = 648.7), the TR gene C2S51_015908 (*BBX32*, K = 520.1), and the PK gene C2S51_011833 (*SELMODRAFT_444075*, K = 652.24), which were selected as the central genes of the MElavenderblush2 module (See **Supplementary Table S9** for the complete list of hub genes and connectivity scores).

**Figure 9:**
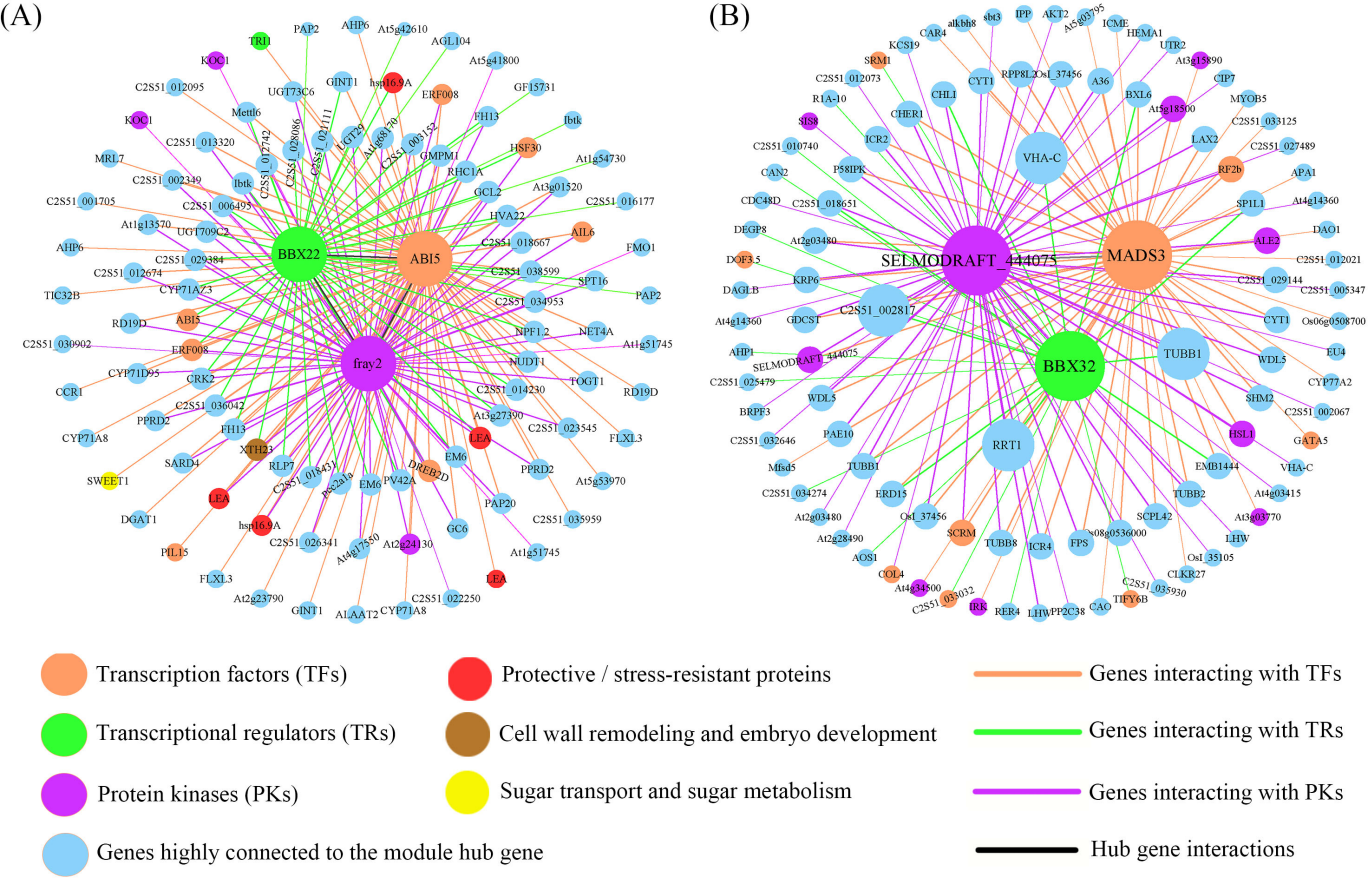
Co-expression network constructed by hub genes. **(A)** Regulatory relationships of hub genes in the MEcoral module. **(B)** Regulatory relationships of hub genes in the MElavenderblush2 module. The figure shows the interactions between hub genes (which are visually highlighted in bold and colored nodes) and other genes. Nodes represent individual genes or proteins, and their colors and shapes indicate their functional roles (e.g., transcription factors, transcriptional regulators, protein kinases, protective/stress-resistant proteins, and those involved in sugar transport and metabolism). The interaction strength between nodes is reflected by the thickness of the lines, with thicker lines representing stronger interactions. The colors of the lines correspond to the types of interactions. The genes or proteins in the figure are detailed in **Supplementary Tables S10** and **S11**.

Based on the three central genes of each module, we constructed the TF-TR-PK co-expression network (**Figure 9**). In the MEcoral module network, 7 TFs, 2 TRs, and 4 PKs were identified (**Figure 9A**; **Supplementary Table S10**). *ABI5* forms a relatively independent yet extendable regulatory hub in the network. On one hand, *ABI5* forms a small-scale “individual” interaction subnetwork with *SWEET1* (a sugar transport-related gene), *TRI1*, *PLI5*, and *LEA* (C2S51_011180). On the other hand, *ABI5* connects with *fray2* (purple diamond, PK) through *LEA* (C2S51_036131), *hsp16.9A* (C2S51_009537), *AIL6* (TF), *ERF008* (C2S51_032164; TF), *At2g24130* (PK), and other functional genes, further expanding its regulatory range in the entire network. Notably, *fray2* also forms a separate interaction cluster with two TRs and various functional genes. Additionally, *BBX22* connects with *ABI5* through *hsp16.9A* (C2S51_013660), and then connects with *fray2* via *HSF30*, forming a complete MEcoral module network.

In the gene co-expression network of the MElavenderblush2 module (**Figure 9B**; **Supplementary Table S11**), *MADS3* (orange) acts as a transcription factor and is one of the core nodes of the entire module. It connects with receptor-like kinases (RKs) such as *At3g03770*, *At4g34500*, *IRK*, and other functional genes, further linking to other transcription factors (*SCRM, RF2b*), protein kinases (*HSL1*, *ALE2*, *At5g18500*), and functional genes (*CYT1*, *LAX2*, *TUBB*, etc.), collectively affecting *SELMODRAFT_444075* (purple), forming an in-depth signaling and transcriptional regulation pathway. Moreover, *MADS3* also connects with *BBX32* (green) via stress or development-related genes such as *ERD15, EMB1444, BXL6*, and *SP1L1*, constructing a multi-level collaborative network from transcription factors to transcriptional regulators and protein kinases. Additionally, functional genes such as *RRT1*, *COL5*, *VHA-C*, and *TUBB1* interact with *MADS3*, *SELMODRAFT_444075*, and *BBX32*, further emphasizing their role as “cross-bridges” in this network. *BBX32* itself interacts unilaterally or bilaterally with several transcription factors (such as *DOF3.5*, *C2S51_033032*, *SRM1*) and other functional genes, and connects with *SELMODRAFT_444075* through three functional genes, C2S51_018651 (transmembrane protein), *CHLI*, and *LHW*, allowing BBX32 to integrate into the network center and form an interaction loop with *MADS3* and *SELMODRAFT_444075*. Notably, *SELMODRAFT_444075* is not only connected with multiple functional genes but also directly associates with a receptor kinase (*SIS8*) and a transcription factor (*COL4*), playing a key role in signal transduction, developmental regulation, and other aspects.

## 4 Discussion

DT is gradually acquired during seed development. As the seed develops, the traits associated with DT change significantly, and the DT within the embryo increases after seed maturation. This study comprehensively analyzed various indicators of perilla seeds, including seed vigor, phenotypic traits, tissue structure, moisture content, and thousand-seed weight. Additionally, WGCNA analysis revealed the changes in traits and gene responses during the DT acquisition process of perilla seeds.

### 4.1 Acquisition of DT during the developmental process

The study found that seed vigor is closely related to changes in phenotypic traits. Between D17 and D22, as the seed embryo’s volume increases, the seed coat becomes more substantial, and the endosperm degenerates; seed vigor gradually increases. Meanwhile, seed moisture content gradually decreases, particularly between D17 and D27, during which moisture drops significantly. This is accompanied by cell expansion and the accumulation of storage substances. Moisture reduction is a key indicator of seeds entering the DT stage (Hell et al., 2019). During the period between D17 and D27, the thousand-seed weight shows a decrease in fresh weight and a continuous increase in dry weight, indicating that seeds are in the critical window for acquiring DT. The change in seed mass is directly related to the acquisition of DT (Martínez-López et al., 2020). Furthermore, changes in the internal tissue structure of the seed, particularly the complete differentiation of the hypocotyl meristem and the peak of storage substance accumulation at D17, mark the seed’s entry into the maturation stage of acquiring DT. The tissue staining results of this study are consistent with these findings, further confirming the important role of hypocotyl meristem differentiation and storage substance accumulation in the acquisition of DT. The comprehensive analysis indicates that the various indicators of perilla seed development are interrelated and coordinated, collectively driving the adaptive transformation of the seed in terms of morphology, structure, and physiology, ultimately enabling the seed to successfully acquire DT.

### 4.2 Physiological and genetic synergistic regulatory mechanisms in the process of acquiring DT

The formation of DT in perilla seeds is a dynamic process driven by the coordinated action of physiological metabolism and gene expression (Sahu et al., 2017). Specifically, the activities of antioxidant enzymes (SOD, POD, CAT) gradually increase during development with the acquisition of DT (**Figure 2**), thereby clearing ROS and alleviating oxidative stress (Almeselmani et al., 2006). The content of the membrane lipid peroxidation product MDA increases after D27, reflecting the dynamic balance of membrane lipid peroxidation. Sugar transport and metabolism (STSM) provides the key material and energy foundation for the acquisition of DT. The expression regulation of *SWEET* and *SUT* genes facilitates the rapid accumulation of sucrose after D22 (**Figure 4**), which acts as an osmoprotectant and signaling molecule, further inducing ABA synthesis genes (Jian et al., 2016; Mathan et al., 2021). Subsequently, ABA content peaks during the D27-D32 period (**Figure 2E**), and the expression levels of its receptor gene *PYL* and transcription factor *ABI5* are also upregulated, forming a positive feedback loop between sugar and ABA (Zhao et al., 2020). Concurrently, the GA degradation gene *GA2O* gradually reduces its influence on GA content during development, relieving the suppression of GA on seed maturation (Bizouerne et al., 2021). This strengthens the ABA-dominated dehydration response. This “ABA increase – GA decrease” hormonal antagonism, coupled with sucrose accumulation, lays an important foundation for the acquisition of DT during seed development.

In terms of protective/stress response proteins (PSR), the expression of *LEA* and *HSP* genes plays a key role in alleviating oxidative damage during the dehydration process. The upregulation of *LEA* genes between D27-D32 (**Figure 4**) results in the production of hydrophobic proteins that can bind to membrane phospholipids, reducing MDA accumulation (**Figure 2F**). These proteins thereby play a protective role in membrane system remodeling (Xu et al., 2020). *HSP* genes are responsible for maintaining protein conformation stability and protecting cell function (Meares et al., 2010). The dynamic changes in these genes not only directly affect the integrity of the membrane system but also reflect the strategy of perilla seeds relying on refined gene regulation to stabilize membranes during the later stages of dehydration. Additionally, aquaporins and seed storage protein DEGs in water balance and storage protein categories regulate the water potential balance within cells and maintain cellular structure integrity during dehydration, preventing damage caused by seed volume shrinkage during dehydration (Padilla-Chacón et al., 2024; Byrt et al., 2023). This further supports the key role of these genes in DT. The different expression patterns of genes related to cell wall remodeling and embryo development (CWR-ED) during development provide flexible structural regulation for seeds under dehydration stress. Expansin genes can increase cell wall extensibility to buffer volume shrinkage (Suslov et al., 2015), while XTH genes enhance cell wall cross-linking to increase wall rigidity (Yamaguchi et al., 2023). Furthermore, CES genes reduce cellulose deposition, thereby preventing excessive shrinkage (Wakabayashi et al., 2023). These physiological and molecular-level systemic regulations together shape the comprehensive adaptive ability of perilla seeds to withstand dehydration during development.

### 4.3 Functional analysis of key modules in the DT acquisition process

WGCNA analysis helps identify genes with similar expression patterns through modularization, revealing genes potentially associated with biological processes. This technology has been successfully applied in wheat drought research (Luo et al., 2019).

The WGCNA analysis used in this study integrates physiological and biochemical indicators with large-scale transcriptomic data to identify dehydration-specific gene modules (**Figure 6**). Enrichment analysis of the MEcoral module indicates that genes within this module play a key role in responding to environmental stresses (such as temperature and water stress) by helping to maintain membrane integrity and antioxidant homeostasis. These processes contribute to preserving membrane integrity and antioxidant homeostasis through lipid remodeling and protein folding protection, thereby enhancing seed DT capacity (Grundy et al., 2015; Barajas-López et al., 2020).

Unlike the MEcoral module, which focuses on membrane homeostasis, enrichment analysis of the MElavenderblush2 module reveals genes primarily involved in photosynthesis, carbon fixation, and starch and sucrose metabolism pathways (**Figures 8B, D**). These pathways provide the necessary energy and carbon source reserves for seed stress resistance and establish a foundation for the synthesis of osmotic regulators. Carbohydrates (such as sucrose), functioning as osmotic protectants and energy storage molecules, are closely associated with photosynthetic activity during seed maturation (Stepanova et al., 2024). We also observed that genes related to “photosynthesis” and “carbon fixation” functions are suppressed during seed development. This is consistent with observations in soybean seeds where photosynthetic function gradually weakens during later maturation stages (Borisjuk et al., 2005). These findings suggest that photosynthesis-related pathways may contribute to osmotic regulation through carbon metabolism products, thereby minimizing dehydration damage during seed development.

Notably, ABA from the MEcoral module and GA from the MElavenderblush2 module interact synergistically and show significant enrichment in plant hormone signal transduction pathways (**Figure 8E**). We found that during DT acquisition in perilla seeds, GA binding to the *GID1* receptor activates the downstream *DELLA* proteins, which display distinct expression patterns. This may correspond to the dynamic control mediated by GA through *DELLA* during Arabidopsis development (Tyler et al., 2004). The interaction between the GA-*GID1* complex and *DELLA* is crucial for *DELLA* protein degradation via the ubiquitin-proteasome pathway (Huang et al., 2024). *DELLA* protein degradation relieves its inhibition of the TF (*PIF3/4*) (Wang et al., 2025), and upon activation, *PIF3/4* shows significant upregulation or downregulation during the complete DT acquisition stage in seeds (D27-D32), thereby promoting perilla seed germination (Sharma et al., 2023). Unlike the GA signaling pathway, the ABA signaling pathway typically functions in plant responses to environmental stress, particularly under drought conditions (Liu et al., 2022). During perilla seed development, expression of the *PYR/PYL* receptor (C2S52_005301) is significantly upregulated at the onset of DT acquisition (D17), then gradually decreases as seeds mature. Other genes show significant upregulation during the complete DT acquisition phase (D27-D32), which may reflect an increased demand for ABA signal perception during seed development (Cui et al., 2020). In ABA signal transduction, *PP2C* phosphatases dephosphorylate *SnRK2* kinases, inhibiting their activity (during D27-D32), thereby negatively regulating ABA signaling (Lin et al., 2021). Concurrently, *SnRK2* kinases interact with ABF (*GBF4*, *ABI5*), regulating their DNA-binding activity and transcriptional regulatory capacity. This interaction can regulate the expression of ABA-responsive genes in Arabidopsis (Fujita et al., 2013).

Based on key module enrichment analysis results, this study demonstrates the coordinated role of ABA and GA during seed DT acquisition. This cross-regulatory mechanism may enhance desiccation resistance during seed development by maintaining membrane and antioxidant homeostasis, while simultaneously ensuring a sustained supply of energy and osmotic regulators, thereby supporting subsequent storage and germination.

### 4.4 Regulatory function of hub genes

Based on the above analysis, this study employs hub gene co-expression networks to clarify the synergistic roles of the MEcoral and MElavenderblush2 co-expression modules in “ABA-GA dynamic balance” and “carbon metabolism-redox homeostasis coupling,” thereby further investigating the mechanisms underlying the DT responses in perilla seeds.

The results show that MEcoral is significantly correlated with ABA, CAT, POD, SS, and Suc (**Figure 6C**). Within this module, the co-expression network comprising key genes such as *ABI5*, *BBX22*, and *fray2* regulates osmotic protection and antioxidant defense through the ABA signaling pathway (**Figure 9A**). *ABI5* is a key transcription factor in the ABA pathway, crucial for seed maturation and stress adaptation (Finkelstein and Lynch, 2000). It functions within the ABA signaling network by activating genes containing ABA response elements (ABREs), such as LEA proteins and sugar transport proteins (Lopez-Molina et al., 2001). Studies have shown that *ABI5* upregulates sugar transport genes (*SWEET1*) and LEA proteins, promoting sucrose accumulation and maintaining membrane integrity, which are crucial for DT (Zinsmeister et al., 2016; Mathan et al., 2021). The role of LEA proteins in dehydration protection has been well-established in species such as cotton (Magwanga et al., 2018), further supporting their key role in perilla seed DT. *ABI5* regulation is controlled by other transcription factors (such as *ABI3* and *ABI4*) and post-translational modifications (such as phosphorylation and ubiquitination), ensuring precise control of stress responses (Liu and Stone, 2014). This further reinforces the central role of *ABI5* in protecting seeds during dehydration through regulated gene expression.

The BBX family is associated with light signals, photomorphogenesis, and abiotic stress responses (Gangappa and Botto, 2014), and plays a key role in linking the ABA signaling pathway with heat stress responses. *BBX22* is a B-box domain transcription factor that directly binds to stress-responsive gene promoters and enhances cellular protection by inducing antioxidant production and coordinating DNA repair responses (Job et al., 2025). Mechanistically, *BBX22* modulates desiccation tolerance by promoting accumulation of protective compounds including ascorbic acid and proline, which overlap with ABA-mediated osmoregulation pathways (Chang et al., 2011), while also preventing DNA damage accumulation that would compromise seed viability during dehydration (Job et al., 2025). *BBX21*, as another member of this family, negatively regulates ABI5 in Arabidopsis, suggesting a complex regulatory relationship within the BBX family (Xu et al., 2014). In this study, *BBX22* functions as a central node, connecting *hsp16.9A*-*ABI5* and *HSF30*-*fray2* (**Figure 9A**), indicating its involvement in both ABA-dependent dehydration responses and heat stress tolerance (Sarkar et al., 2014; Hahn et al., 2011). Heat shock proteins (HSPs) are key factors in heat tolerance, and their interaction with the ABA signaling pathway is supported by research on *HSFA6b* in Arabidopsis, indicating that *HSFA6b* links ABA signaling with heat tolerance (Huang et al., 2016). The role of *BBX22* as a molecular bridge between ABA and heat stress signals aligns with the BBX family’s function in integrating multiple stress signals (Talar and Kiełbowicz-Matuk, 2021), expanding the heat stress response network in perilla seeds. This dual role of *BBX22* has not been fully explored in previous studies, and our findings provide new evidence for its integrative role in stress signaling.

On the other hand, MElavenderblush2 is significantly correlated with GA, Fru, Glu, MC, and MDA (**Figure 6C**). The GA signaling pathway regulates photosynthesis and monosaccharide metabolism, providing energy and osmotic substances during the early stages of dehydration in the developmental process (Liu and Hou, 2018). *MADS3*, as a MADS-box transcription factor, is involved in the GA signaling pathway and regulates developmental processes such as flowering time and fruit development (Ng and Yanofsky, 2001). The repression of *MADS3* during late seed maturation is advantageous for desiccation tolerance because its continued expression creates developmental conflicts, preventing the coordinated metabolic shutdown and developmental transitions required for proper seed desiccation, as demonstrated by delayed desiccation kinetics in *AGL15*/*MADS3* overexpression lines (Fang and Fernandez, 2002; Perry et al., 1996). This repression is mediated by epigenetic silencing mechanisms involving *HSI2*/*VAL1*, which ensures the timely transition from active maturation programs to the quiescent state necessary for optimal desiccation tolerance (Chen et al., 2018). Studies have shown that *MADS3* interacts with receptor kinases (such as At3g03770, At4g34500) and genes including *IRK*, *LAX2*, *TUBB*, and *CYT1*, promoting monosaccharide accumulation and vesicle transport in the early stages of dehydration while coordinating plant cell communication (Dong et al., 2019; Hailemariam et al., 2024). *MADS3*, together with other transcription factors or regulatory proteins, regulates floral organ development in Arabidopsis and rice (Li et al., 2011; Kaufmann et al., 2009). The role of *MADS3* in stress responses is consistent with its function in other species. For example, *OsMADS26* in rice negatively regulates pathogen resistance and drought tolerance, indicating its role in stress signaling (Khong et al., 2015). The interaction between *MADS3* and light signaling factors such as *BBX32* further increases the complexity of stress response regulation in perilla seed DT acquisition, suggesting that *MADS3* and *BBX32* may coordinate GA and light signaling pathways to optimize energy metabolism and stress adaptation.

**Figure 10:**
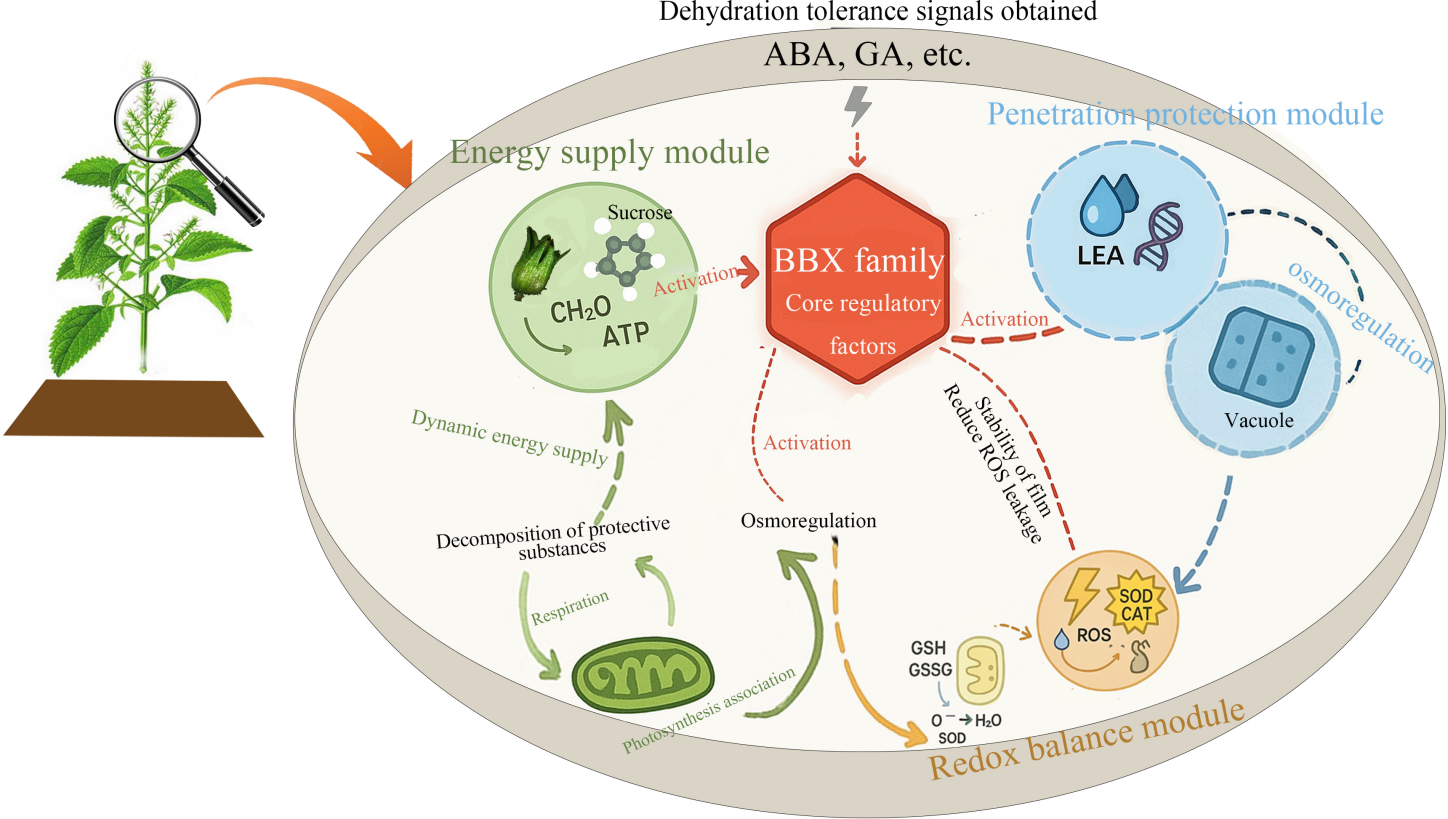
The hypothesized regulatory pathway for DT acquisition in perilla seeds. The diagram shows three main modules: the energy supply module (green), which provides dynamic energy through processes such as respiration and photosynthesis; the osmotic protection module (blue), which stabilizes the cell membrane and osmotic regulation through LEA proteins and vacuoles; and the redox balance module (orange), which defends against oxidative stress through antioxidants such as superoxide dismutase (SOD). These processes are regulated by desiccation tolerance signals (ABA and GA) and core regulatory factors from the BBX family.

Based on these findings, we propose a novel regulatory network model in which the BBX family plays a central role within perilla seed DT network (**Figure 10**). This model not only highlights the BBX family’s broad involvement across multiple regulatory modules but also lays the foundation for future investigations into its mechanistic role in DT. Although this study did not directly validate the function of key genes, we suggest that future research should focus on functional validation of genes such as *ABI5* and *BBX22*. This could be achieved through advanced techniques such as CRISPR-Cas9-based gene editing or overexpression studies, which would provide a deeper understanding of how these genes contribute to the molecular mechanisms underpinning DT. Such work will not only advance our knowledge of plant stress responses but also provide insights into potential targets for crop improvement strategies aimed at enhancing drought resistance.

## 5 Conclusion

This study reveals for the first time the dynamic mechanism of DT acquisition in perilla seeds during development and elucidates the network framework of coordinated regulation involving morphological structure, physiological metabolism, and gene expression. Our findings indicate that the vigor of perilla seeds gradually increases as the embryo enlarges and the seed coat thickens, while the moisture content significantly decreases during D17-D27, marking the transition of the seeds into the DT acquisition phase. Physiologically, the increased activity of antioxidant enzymes and the dynamic balance of MDA together maintain oxidative homeostasis. Furthermore, sucrose accumulation and the antagonistic effect of ABA and GA regulate osmotic protection and dehydration response genes, thereby preventing dehydration damage during seed DT acquisition. WGCNA analysis further reveals that the MEcoral module maintains membrane integrity through lipid metabolism, endoplasmic reticulum protein processing, and the ABA signaling pathway, while the MElavenderblush2 module regulates energy supply and cell wall remodeling through photosynthetic carbon metabolism and GA signaling. The co-expression network of key hub genes (such as *ABI5*, *BBX22*, *MADS3*) indicates that BBX family genes may coordinate antioxidant defense, and energy metabolism by integrating ABA, heat stress, and light signaling pathways, ultimately enabling seeds to withstand dehydration during the DT acquisition process. This study provides a new perspective for understanding the molecular mechanisms of seed DT and lays the theoretical foundation for genetic improvement of crop stress resistance.

## Data Availability

The datasets generated and/or analyzed during the current study are available in the NCBI SRA repository, with accession number PRJNA1256419 (https://www.ncbi.nlm.nih.gov/sra/PRJNA1256419). All data generated or analyzed during this study are included within the article and its additional files.
