# Supplementary material for: ABA-GA antagonism and modular gene networks cooperatively drive acquisition of desiccation tolerance in perilla seeds
Source: Front Plant Sci. 2025 Jul 23;16:1624742. doi: 10.3389/fpls.2025.1624742 (PMC12325373; doi:10.3389/fpls.2025.1624742)
Supplement: Supplementary file 1 [file DataSheet1.docx]

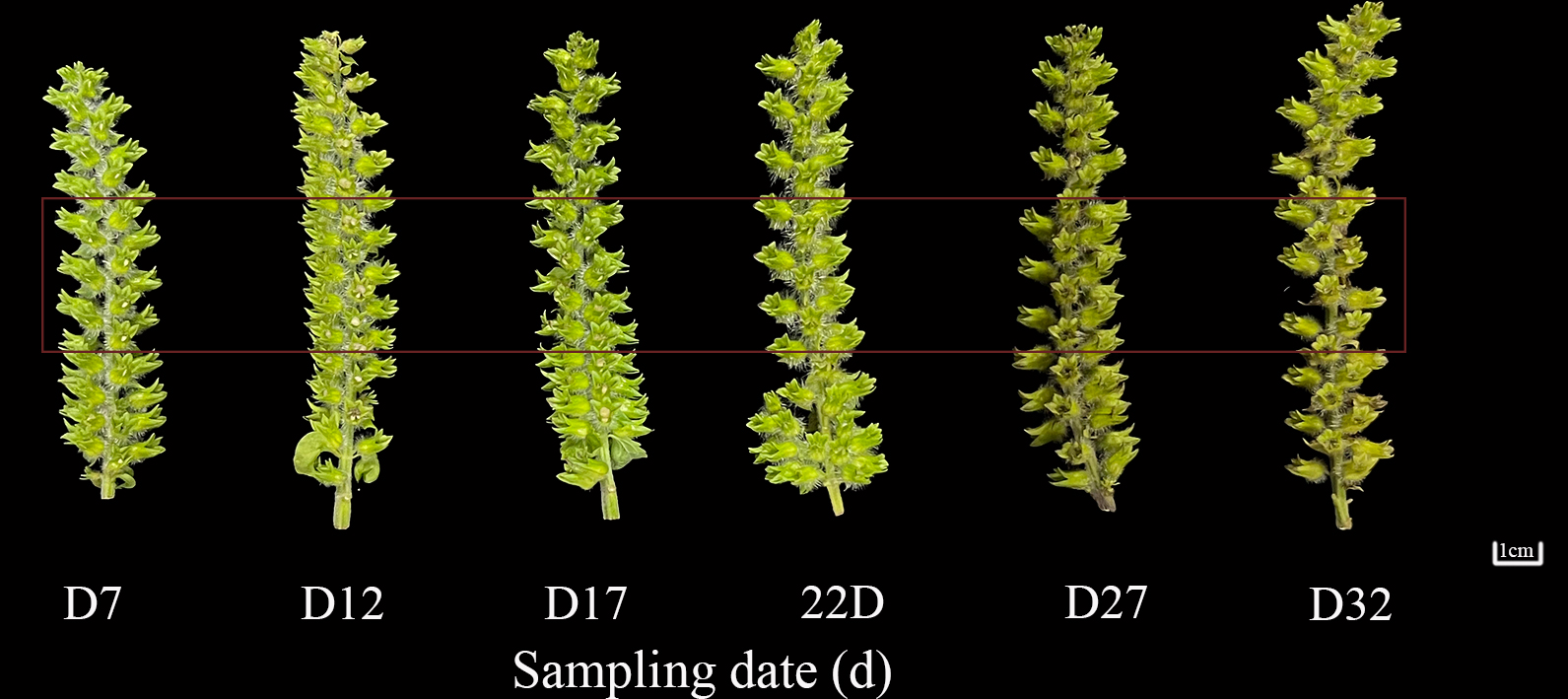


Fig. S1 Morphological changes of the plant over time, with samples collected at different days (D7, D12, D17, D22, D27, and D32). The horizontal red bar marks the collected portion.


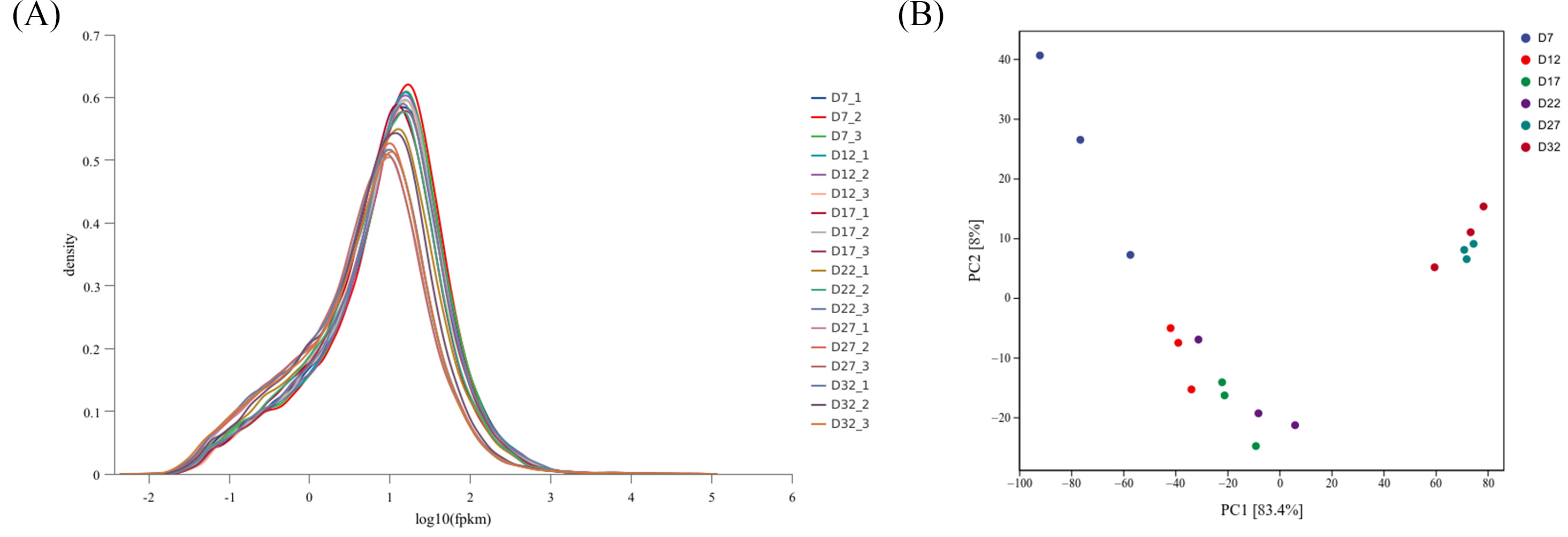


Fig. S2: (A) Density plot of log-transformed read counts (log10(fpkm)) across different time points (D7, D12, D17, D22, D27, D32) and biological replicates (1, 2, 3). The density distribution indicates the variation in read counts at each time point. (B) Principal component analysis (PCA) of the log-transformed data showing the separation of samples along principal components (PC1 and PC2).


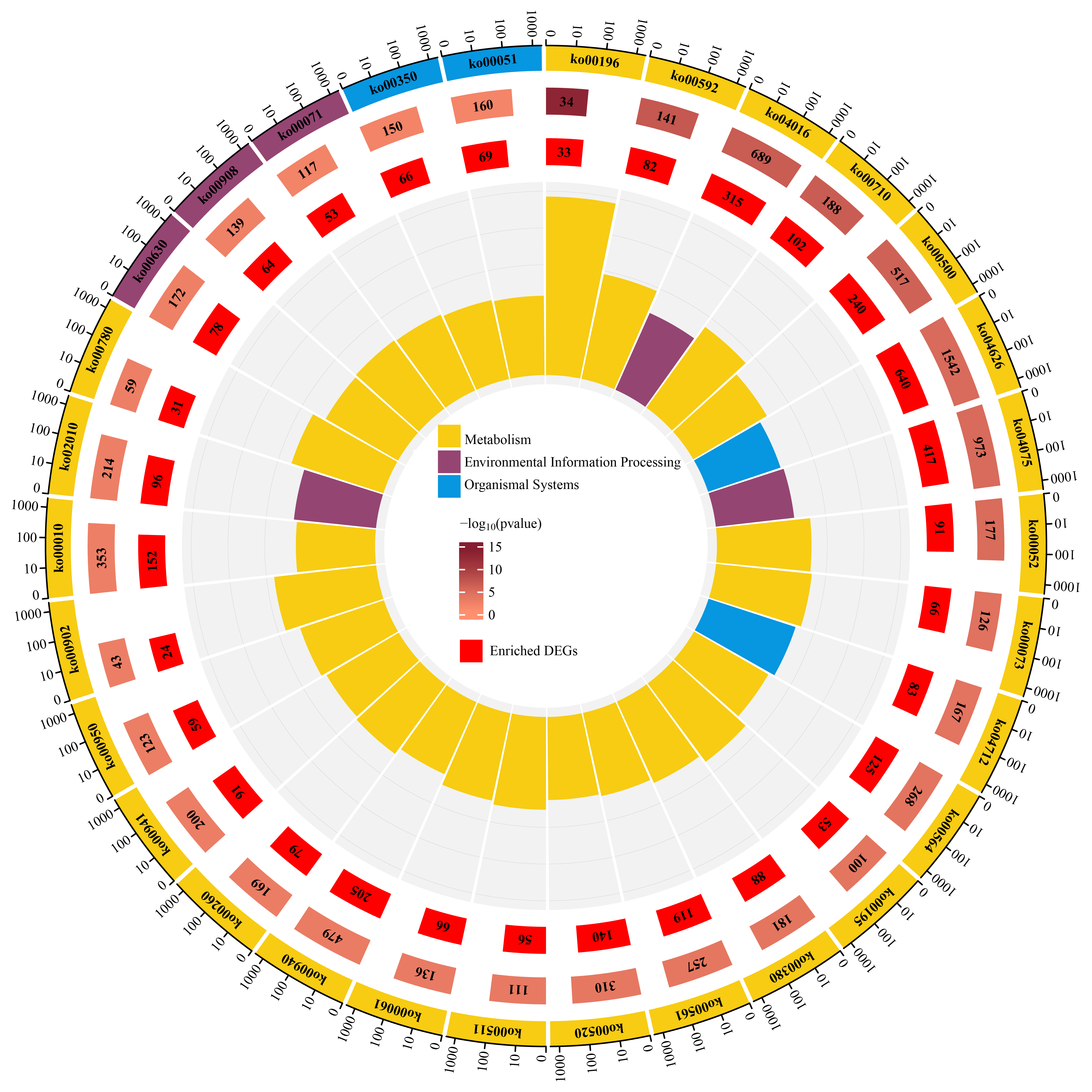


Fig. S3 KEGG pathway enrichment results. The outer ring represents KEGG pathway categories, which are divided into three categories: metabolism, environmental information processing, and organism systems, with each category distinguished by a different color. The second ring represents the significance of enriched pathways, with the color indicating changes in -log10(p-value), where deeper red shading indicates pathways with stronger statistical significance. The outermost ring displays the number of DEGs enriched in the pathways, shown in red.


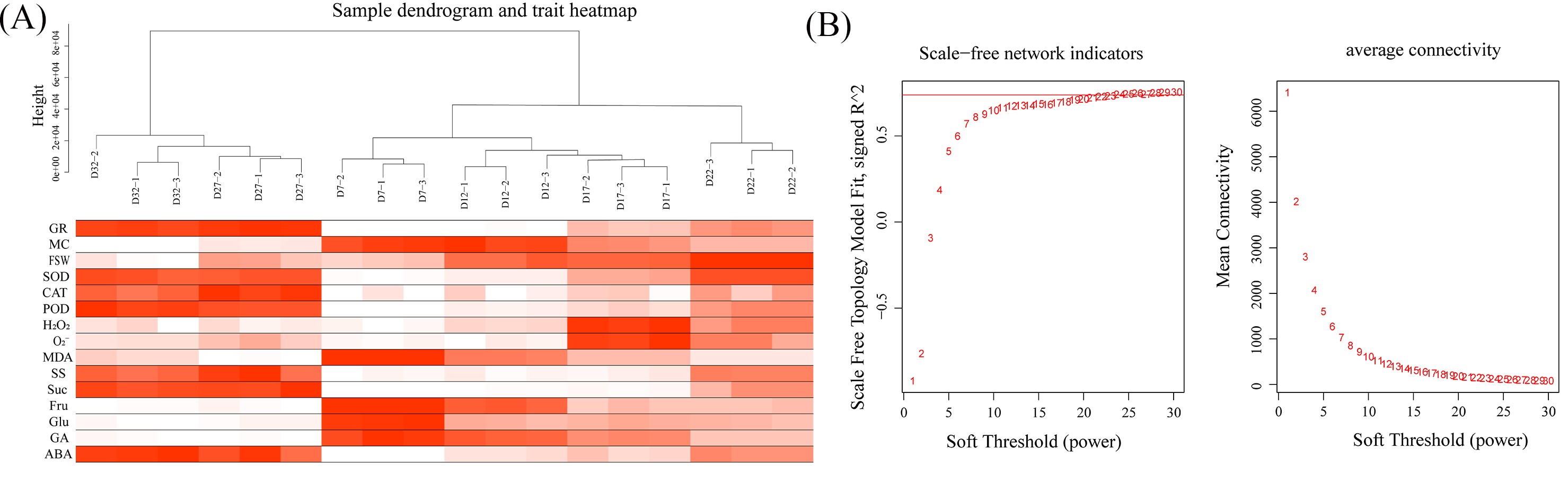

Fig S4 (A) The sample dendrogram and trait heatmap display the expression patterns of different samples across multiple traits. In the heatmap, red and white indicate the correlation between different time points and traits. (B) The scale-free network metrics in the network analysis show the trend of model fitting (left) and average connectivity (right) under different soft thresholds.
